# Cellular responses to ErbB-2 overexpression in human mammary luminal epithelial cells: comparison of mRNA and protein expression

**DOI:** 10.1038/sj.bjc.6601458

**Published:** 2004-01-06

**Authors:** S L White, S Gharbi, M F Bertani, H-L Chan, M D Waterfield, J F Timms

**Affiliations:** 1 Ludwig Institute for Cancer Research, Wing 1.1, Cruciform Building, Gower Street, London WCIE 6BT, UK

**Keywords:** ErbB-2, breast cancer, microarray, 2D-DIGE, interferon

## Abstract

Microarray analysis offers a powerful tool for studying the mechanisms of cellular transformation, although the correlation between mRNA and protein expression is largely unknown. In this study, a microarray analysis was performed to compare transcription in response to overexpression of the ErbB-2 receptor tyrosine kinase in a model mammary luminal epithelial cell system, and in response to the ErbB-specific growth factor heregulin *β*1. We sought to validate mRNA changes by monitoring changes at the protein level using a parallel proteomics strategy, and report a surprisingly high correlation between transcription and translation for the subset of genes studied. We further characterised the identified targets and relate differential expression to changes in the biological properties of ErbB-2-overexpressing cells. We found differential regulation of several key cell cycle modulators, including cyclin D2, and downregulation of a large number of interferon-inducible genes, consistent with increased proliferation of the ErbB-2-overexpressing cells. Furthermore, differential expression of genes involved in extracellular matrix modelling and cellular adhesion was linked to altered adhesion of these cells. Finally, we provide evidence for enhanced autocrine activation of MAPK signalling and the AP-1 transcription complex. Together, we have identified changes that are likely to drive proliferation and anchorage-independent growth of ErbB-2- overexpressing cancer cells.

The receptor tyrosine kinase ErbB-2 (Her-2/Neu) is overexpressed in 25 – 30% of all human breast cancers, and is associated with a poor prognosis and an increased likelihood of metastasis (Slamon *et al*, 1987). However, despite intensive research efforts, the biological mechanisms underlying the oncogenicity of ErbB-2 are still poorly understood. ErbB-2 is a member of the ErbB family of growth factor receptors comprising EGFR (ErbB-1), ErbB-2, ErbB-3 and ErbB-4. Exposure of cells to ErbB receptor-specific ligands results in receptor homodimerisation and/or cross-family heterodimerisation, kinase activation, and self- and crossphosphorylation of cytoplasmic tyrosine residues. Various adaptor molecules bind to the phosphorylated receptors, resulting in signal transduction initiation that ultimately regulates gene transcription (Yarden and Sliwkowski, 2001). It appears that the relative expression level of each ErbB family member, as well as ligand specificity, determines the nature of the dimerisations, and hence the repertoire of adaptors which bind to the activated receptors. This in turn determines the specificity and strength of downstream signalling (Yarden and Sliwkowski, 2001). While ErbB-2 has no known ligands, it is the preferred heterodimerisation partner of EGFR, ErbB-3 and ErbB-4 (Graus-Porta *et al*, 1997). Thus, ErbB-2 overexpression is believed to enhance signalling from these receptors in response to binding of their specific ligands, but also independently through forced receptor association.

Although ErbB-2 overexpression alone cannot transform cells (Ross and Fletcher, 1998), it has highly integrated roles in cellular signalling that impact on the classic ‘hallmarks’ of cancer (Hanahan and Weinberg, 2000). Indeed, ErbB-2 overexpression can provide self-sufficiency in growth signals, enhanced survival, anchorage-independent growth, and, importantly, promote tissue invasion and metastasis. An example of the signalling interplay that exists is the interaction of ErbB family members with integrins, the major receptors for the extracellular matrix (ECM) (Eliceiri, 2001). Indeed, growth factor-induced proliferation and migration are dependent on integrin-mediated adhesion to the ECM, including the ECM component fibronectin (FN1) (Danen and Yamada, 2001).

DNA microarrays allow the parallel analysis of thousands of genes and offer a powerful technology to further our understanding of breast cancer. Global gene-expression profiling has been applied to breast carcinomas for diagnostic and prognostic purposes, and has been used to compare breast cancer cell lines (Ross *et al*, 2000), tumour tissues (Perou *et al*, 1999), and to classify breast cancer tissue and cell lines into subgroups according to oestrogen receptor status (Gruvberger *et al*, 2001), physiological variation (Perou *et al*, 2000), underlying hereditary mutations (Hedenfalk *et al*, 2001; van ‘t Veer *et al*, 2002) and invasiveness/metastasis (Zajchowski *et al*, 2001; van ‘t Veer *et al*, 2002). Microarray technology also offers the ability to investigate changes in gene expression resulting from the overexpression of single oncogenes commonly found in breast cancer. Ideally, this involves comparison of primary breast tumour material and normal healthy tissue from the same patient. However, this may not be possible: taking a biopsy of healthy tissue may be ethically and clinically unsatisfactory; samples may have different proportions of mixed cell types, and, depending on the grade and stage of the carcinoma, the tumour sample may have secondary mutations resulting from chromosomal instability, making the results of a microarray analysis hard to interpret. While cell lines provide a more genetically homogenous choice than tissues for breast cancer studies, they should be closely matched and ideally be human mammary luminal epithelial cells (HMLECs), the cell type from which the most common form of breast cancer, infiltrating ductal carcinoma (IDC), arises.

Despite the increasing number of reports using expression profiling to decipher the molecular mechanisms of cancer, or to predict clinical outcome or survival, these studies rarely address the correlation between mRNA and protein expression. Indeed, it is as yet unknown whether changes observed at the mRNA level are translated into similar changes at the protein level on a global scale, or indeed whether such ‘linked’ mRNA and protein changes are relevant to the onset of cancer. The correlation between mRNA and protein expression may be very low over the entire genome due to the effects of differential mRNA stability gene-specific regulation of translation, mRNA silencing and differential protein stability. However, parallel proteomic and microarray comparisons are still extremely useful if a cell's response to a given stimuli or oncogenic conversion are to be properly deciphered. Such comparisons offer the potential to understand the nature of deregulated gene expression that leads to cancer progression.

Here we describe the results of a microarray analysis that profiles the transcriptional responses to ErbB-2 overexpression in a model HMLEC system, HB4a and C3.6 (Harris *et al*, 1999). This model is ideal for the microarray analysis of the effect of ErbB-2 on gene expression in breast cancer progression, as it consists of the cell type from which IDCs of the breast arise, has an ErbB-2 expression level comparable to that seen in carcinomas, and is comprised of a closely matched test and reference sample. Using this model cell system and stimulation with heregulin *β*1 (HRG*β*1), we have specifically investigated the transcriptional changes that occur downstream of ErbB-2/3 heterodimerisation and signalling. Moreover, we have correlated some of the observed mRNA changes with changes at the protein level in a parallel proteomics study, and related some of these changes to the transformed characteristics of these cell lines, specifically enhanced autocrine signalling, proliferation/cell cycle progression and cellular adhesion.

## MATERIALS AND METHODS

### Cell lines, tissue culture, mitogen stimulation and RNA extraction

Generation of the parental HB4a mammary luminal epithelial cell line and derivatives overexpressing ErbB2 (C3.6 and C5.2) has been described previously (Harris *et al*, 1999). Cells were cultured in RPMI-1640 media with 10% (v/v) foetal calf serum (FCS), 2 mM glutamine, 100 IU ml^−1^ penicillin, 100 *μ*g ml^−1^ streptomycin, 5 *μ*g ml^−1^ hydrocortisone and 5 *μ*g ml^−1^ insulin (both Sigma, Poole, UK) at 37°C in a 10% CO_2_-humidified incubator. The luminal epithelial cell line Lum878 was maintained under the same growth conditions and derived from normal human mammary luminal epithelial cells by immortalisation with SV40 large T antigen and human telomerase reverse transcriptase (from Dr Michael O'Hare, Ludwig Institute for Cancer Research). The breast tumour cell lines BT474, MCF-7, SKBr3, MDA-MB-361, MB-435 and MB-453 were obtained from the American Type Culture Collection (ATCC), and maintained under culture conditions listed on the ATCC website (http://www.atcc.org/SearchCata logs/Search.cfm). Cell lines SUM159 and SUM185 were from The University of Michigan Breast Cell/Tissue Bank (from Dr Stephen Ethier). Details of these cell lines and culture conditions are found on the SUM-breast cell line web page (http://www.cancer.med.umich.ed u/breast_cell/clines/clines.html). For stimulation experiments, HB4a and C3.6 cells at 50 – 60% confluency were starved for 48 h in RPMI-1640 media with 0.1% FCS, 2 mM glutamine, 100 IU ml^−1^ penicillin, 100 μg ml^−1^ streptomycin and 5 μg ml^−1^ hydrocortisone (serum-starvation media). Cells were then stimulated by the addition of 1 nM HRG*β*1 (R&D Systems) for 1, 4, 8, 12, 18 and 24 h. Four replicate plates of cells were stimulated for each timepoint. Total RNA was isolated from each replicate experiment using TRIZOL™ Reagent (Life Technologies Inc., Gaithersburg, MD, USA), according to the manufacturer's instructions.

### Microarray experiments

Microarray analyses were performed on RNA from each of the four replicate experiments. A ‘reciprocal duplicate’ labelling strategy was used to minimize the effects of dye bias and to provide statistical confidence; two hybridisations were performed with the HB4a sample labelled with Cy3 and the C3.6 sample labelled with Cy5 (duplicates), and two hybridisations were performed with the labelling reversed (reciprocal duplicates). Hver1.2.1 microarrays were obtained from The Wellcome Trust Sanger Institute. Probes consist of a redundant group of 9932 PCR-derived, sequence-verified cDNA features (representing ∼6000 unique genes) spotted onto amine-binding glass slides (details available on The Wellcome Trust Sanger Institute Microarray Facility homepage (http://www.sanger.ac.uk/Projec ts/Microarrays/)). Protocol 5 from the Facility homepage was used for generation of fluorescently labelled cDNA targets using direct Cy dye incorporation. Briefly, 25 *μ*g of RNA was used to produce labelled cDNA by anchored oligo(dT)-primed reverse transcription with Superscript II Reverse Transcriptase (Life Technologies), in the presence of Cy3- or Cy5-dUTP (Amersham, Chalfont, St Giles, UK). Unincorporated dye was removed using Autosequ-50 Columns (Amersham, Chalfont, St Giles, UK) and repetitive DNA sequences were blocked with 8 *μ*g Cot1 (Roche Diagnostic Ltd, Lewes, UK) and 8 *μ*g poly(dA) (Sigma) DNA before hybridisation at 47°C overnight.

### Data analysis and mining

Microarrays were scanned using a ScanArray 4000XL (Packard BioChip Technology, Billerica, MA, USA) according to the manufacturer. The raw image data were acquired using ScanArray v3.0 software, and then exported to QuantArray v3.0 software for data extraction. Features and background areas were defined using QuantArray's Histogram method, and the median intensity for each channel was used to scale dye-emission signals. Features visibly affected by hybridisation artefacts were manually flagged for exclusion. Background subtracted signal intensities of the Cy3 and Cy5 channels for each feature were then loaded into GeneSpring v4.2 (Silicon Genetics, Redwood City, CA, USA) for normalisation. The expression level of each probe was derived by dividing the fluorescence intensity of the C3.6 result by the HB4a result for each probe in the four replicates. Intensity-dependent normalisation was applied, where the ratio was reduced to the residual of the Lowess fit of the intensity *vs* ratio curve (refer to GeneSpring v4.2 documentation for more information). When the control channel value was below 10.0, the data point was considered bad. The final ratio is the geometric mean of the four replicates. Expression differences were considered significant if the ratio of C3.6 : HB4a was 0.5 (downregulated), or ⩾2.0 (upregulated). Probes were annotated with an Ensembl Description and ID (from Ensembl (http://www.ensembl.org/Homo_sa piens/) Release 7.29a2). Genes were classified into broad functional groups based on classifications from the Gene Ontology™ Consortium (Ashburner *et al*, 2000) and literature reviews.

### Differential protein expression

Differential protein expression data were taken from a 2D-difference gel electrophoresis (2D-DIGE) analysis comparing the proteomes of serum-starved HB4a and C3.6. cells (Gharbi *et al*, 2002). This global proteomic analysis provided statistically significant (*P*<0.05) expression differences (as average ratios) for 16 proteins that were present on the microarray. The expression of a further 27 proteins was compared in serum-starved HMLECs by immunoblotting with specific antibodies, using enhanced chemiluminescence for detection, and densitometry for quantification (QuantityOne, BioRad). At least three replicate blots were scanned and quantified for each protein, and the average ratio of C3.6 *vs* HB4a was determined.

### Cell-adhesion assays

Cells were trypsinised, washed twice in serum-free media and seeded into 96-well plates (5 × 10^3^ cells well^−1^) in either serum-free media, or serum-free media plus 1 nM HRG*β*1. Wells were either untreated (adhesion to plastic), or coated with human plasma FN (Sigma) (adhesion to FN). Cells were allowed to adhere for 1 h at 37°C, and then washed with PBS. A volume of 50 *μ*l of 1 mg/ml^−1^ 3-[4,5-dimethyl-thiazol-2-yl]-2,5-diphenyl-tetrazolium bromide (MTT) (Sigma) in serum-free media was added, and cells were further incubated for 4 h. DMSO (100 *μ*l) was added, the plates were shaken for 20 min at 22°C, and the absorbance read at 540 nm. The values given are the average of eight replicate readings.

### Media-swap experiment

Conditioned media (CM) was generated by growth factor treatment of serum-starved HB4a and C3.6 cells (EGF – 1 nM or Hrg*β*1 – 1 nM) for 4 h, followed by extensive washing and replacement with serum-free media for a further 4 h. The conditioned media was used to stimulate serum-starved HB4a and C3.6 cells for 15 min. Mitogenic signalling was monitored by immunoblotting with phosphospecific antibodies to ERK1/2. To test for ErbB-specific signalling, CM media was added to serum-starved cells that had been pretreated with 5 *μ*M AG-1478 (a potent ErbB-kinase inhibitor) for 30 min.

## RESULTS

### Global changes in gene expression

To specifically identify ErbB-2-dependent mRNA changes, ErbB-2-overexpressing C3.6 cells and their normal parental counterparts HB4a were serum-starved and stimulated with the mitogen HRG*β*1 for different times prior to RNA extraction and microarray analysis of relative mRNA levels. In these experiments, the two cell lines were directly compared against one another at each timepoint by cohybridising labelled cDNA from both cell types onto the same array. Heregulin *β*1 stimulation would be expected to signal almost exclusively via ErbB-2/3 heterodimers, given the propensity of ErbB-3 to heterodimerise preferentially with ErbB-2, the inability of HRG*β*1 to stimulate EGFR/ErbB-2 heterodimers (Yarden and Sliwkowski, 2001) and the absence of ErbB-4 in these cells (Harris *et al*, 1999; Timms *et al*, 2002).

**Figure 1:**
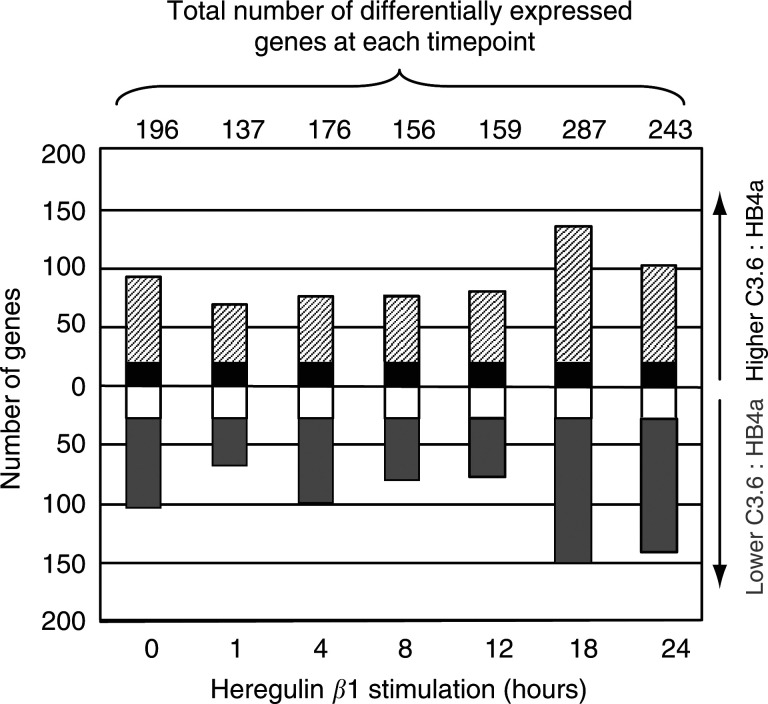
Analysis of global differences in mRNA expression between C3.6 and HB4a HMLECs. Total number of genes with mRNA ratios (C3.6 : HB4a) with a geometric mean of 2.0 or 0.5 in serum-starved cells (0), and in cells at six different times (1, 4, 8, 12, 18, and 24 h) after HRG*β*1 treatment. The hashed area represents 20 genes that are upregulated in C3.6 cells, the gray area, down regulated genes, black and white areas, up and down regulated genes at all time point, respectively.

Of the 9932 probes present on the microarray, 667 probes displayed a greater than two-fold difference in expression between C3.6 and HB4a cells at one or more of the timepoints. The numbers of genes with differential expression did not differ substantially between T1 and T12, but there were significant differences at T0, T18 and T24 (Figure 1

). These variations may reflect the fact that the C3.6 cells display a greater degree of growth-factor independence than the parental cells, and an increased proliferative capacity, progressing through the cell cycle more rapidly in response to mitogenic stimulation, as previously reported (Timms *et al*, 2002).

Genes that are constitutively higher or lower (i.e. at all timepoints) in C3.6 *vs* HB4a cells are expected to have an expression directly affected by ErbB-2 overexpression, and may not be related to mitogen stimulation *per se*. There were 21 such genes upregulated (including ErbB-2) and 27 genes downregulated (Figure 1 and Table 1

**Table 1:** Genes that are constitutively: (a) two-fold upregulated, and (b) two-fold downregulated in C3.6 cells relative to HB4a cells (i.e. under serum-starved conditions and at all timepoints after stimulation with 1 nM HRG*β*1).

**Abbrev**	**Ensembl Number and description**	**Function**
	*Constitutively upregulated*	
S100P	ENSG00000163993 S-100P PROTEIN	CA binding
CPS1	ENSG00000021826 CARBAMOYLPHOSPHATE SYNTHASE	Metabolism
HIBCH	ENSG00000115404 3-HYDROXYISOBUTYRYL-COENZYME A HYDROLASE	Metabolism
C20orf16	ENSG00000088826 POLYAMINE OXIDASE ISOFORM-3	Metabolism
	ENSG00000162424 SUCCINATE DEHYDROGENASE	Metabolism
	ENSG00000130021 GS1 PROTEIN	Metabolism
FDX1	ENSG00000137714 ADRENODOXIN, MITOCHONDRIAL PRECURSOR	Metabolism
COX6C	ENSG00000164919 CYTOCHROME C OXIDASE POLYPEPTIDE VIC PRECURSOR	Metabolism
CCND2	ENSG00000118971 G1/S-SPECIFIC CYCLIN D2	Proliferation
	ENSG00000151465 PROTEIN D123	Proliferation
UCHL1	ENSG00000154277 UBIQUITIN CARBOXYL-TERMINAL HYDROLASE ISOZYME L1 PROTEIN	Processing
ERBB2	ENSG00000141736 RECEPTOR PROTEIN-TYROSINE KINASE ERBB-2 PRECURSOR	Signalling
LCP1	ENSG00000136167 L-PLASTIN (LYMPHOCYTE CYTOSOLIC PROTEIN 1)	Structural protein
VIM	ENSG00000026025 VIMENTIN	Structural protein
KLF12	ENSG00000118922 KRUEPPEL-LIKE FACTOR 12	Transcription
TRIM29	ENSG00000137699 ATAXIA-TELANGIECTASIA GROUP D-ASSOCIATED PROTEIN	Transcription
CPNE3	ENSG00000085719 COPINE III	Transport
SSFA2	ENSG00000138434 SPERM-SPECIFIC ANTIGEN 2	Unknown
	ENSG00000110696 SMALL ACIDIC PROTEIN	Unknown
	ENSG00000077232 CDNA FLJ14741 FIS, CLONE NT2RP3002628	Unknown
	UNIDENTIFIED TRANSCRIPT	Unknown
		
	*Constitutively downregulated*	
SPARC	ENSG00000113140 SPARC PRECURSOR	Ca binding
IFITM1	ENSG00000142089 INTERFERON-INDUCED TRANSMEMBRANE PROTEIN 1	Immune response^*^
WNT5A	ENSG00000114251 WNT-5A PROTEIN PRECURSOR	Ligand
TYMS	ENSG00000080868 THYMIDYLATE SYNTHASE	Metabolism
OAS1	ENSG00000089127 2'-5'-OLIGOADENYLATE SYNTHETASE 1	Metabolism^*^
PAPSS2	ENSG00000148615 3'-PHOSPHOADENOSINE 5′-PHOSPHOSULFATE SYNTHETHASE 2	Metabolism
CYBA	ENSG00000051523 CYTOCHROME B-245 LIGHT CHAIN	Metabolism
FTHFD	ENSG00000144908 10-FORMYLTETRAHYDROFOLATE DEHYDROGENASE	Metabolism
	ENSG00000133700 INTERFERON INDUCED TRANSMEMBRANE PROTEIN	Proliferation^*^
	ENSG00000137440 HEPARIN BINDING PROTEIN PRECURSOR	Proliferation
	ENSG00000162569 UBIQUITIN CROSS-REACTIVE PROTEIN PRECURSOR	Protein Processing^*^
KLK8	ENSG00000160327 NEUROPSIN PRECURSOR	Protein Processing
TIMP3	ENSG00000100234 METALLOPROTEINASE INHIBITOR 3 PRECURSOR (TIMP-3)	Protein Processing
TRIP12	ENSG00000153827 THYROID RECEPTOR INTERACTING PROTEIN 12	Protein Processing
SERPINH1	ENSG00000149257 47 KDA HEAT SHOCK PROTEIN PRECURSOR	Protein Processing
PRSS11	ENSG00000166033 SERINE PROTEASE HTRA1 PRECURSOR	Protein Processing
WFDC2	ENSG00000101443 MAJOR EPIDIDYMIS-SPECIFIC PROTEIN E4 PRECURSOR (HE4)	Protein Processing
C1S	ENSG00000126750 PRECURSOR EC 3.4.21.- SERINE PROTEASE	Protein Processing
IGFBP3	ENSG00000146674 INSULIN-LIKE GROWTH FACTOR BINDING PROTEIN 3 PRECURSOR	Signalling^*^
	ENSG00000101608 MYOSIN REGULATORY LIGHT CHAIN 2, NONSARCOMERIC	Structural Protein
FN1	ENSG00000115414 FIBRONECTIN PRECURSOR (FN)	Structural Protein
FBLN2	ENSG00000163520 FIBULIN-2 PRECURSOR	Structural Protein
ISGF3G	ENSG00000100915 TRANSCRIPTIONAL REGULATOR ISGF3 GAMMA SUBUNIT	Transcription^*^
KDELR2	ENSG00000136240 ER LUMEN PROTEIN RETAINING RECEPTOR 2	Transport
	ENSG00000136193	Unknown
IFIT1	ENSG00000152777 IFN-INDUCED PROTEIN WITH TETRATRICOPEPTIDE REPEATS 1	Unknown^*^

Functional classifications (‘Function’) were subjectively given, based on classifications from the Gene Ontology™ Consortium (Ashburner *et al*, 2000), and literature reviews. IFN-inducible genes are denoted with an asterisk.

). When classified into groups based on functional ontology, approximately one-third of the upregulated genes were found to be involved in cellular metabolic processes. For example, carbamoylphosphate synthetase 1 (CPS1), the most highly expressed gene in the data series, is part of the mitochondrial urea cycle. While differential expression of metabolic enzymes has been reported in breast cancer (Hennipman *et al*, 1988), it is possible that this upregulation occurs as a secondary consequence of the increased energy requirements of hyperproliferating cells, rather than of a transformed phenotype (Guppy *et al*, 2002).

Several of the upregulated genes are reported to have increased expression in breast cancer. These include: the calcium-binding protein S100P (Guerreiro Da Silva *et al*, 2000); the structural protein L-plastin (LCP1) (Lin *et al*, 1988) and the intermediate filament protein vimentin, the upregulation of which has been correlated with increased invasiveness (Zajchowski *et al*, 2001). However, the constitutive upregulation of the cell cycle regulator cyclin D2 (CCND2) is a novel finding, and of particular interest, given that ErbB-2 overexpression increases cyclin-dependent kinase (cdk) activity in these cells (Timms *et al*, 2002). Notably, of the 27 constitutively downregulated genes, seven are interferon- (IFN) inducible (indicated with an asterisk in Table 1). Of these, the downregulation of the transcriptional regulator ISGF3 gamma subunit (ISGF3G) has significant implications. ISGF3G, together with signal transducer and activator of transcription 1 (STAT1) and STAT2, forms the ISGF3 transcription complex, which is required for transcription of IFN*α/β*-inducible genes, including IFN*β* itself (Nakaya *et al*, 2001). Thus, decreased ISGF3G expression in the C3.6 cells may cause the lowered transcription of the other IFN-inducible genes.

A number of constitutively downregulated genes have reported roles in cellular adhesion processes. These include: fibronectin 1 (FN1), a major ECM component required for integrin-mediated signalling (reviewed in Danen and Yamada, 2001); fibulin 2 (FBLN2), which colocalises with FN1 *in vivo* (Gu *et al*, 2000); and the tissue inhibitor of metalloproteinase 3 (TIMP 3), which is involved in regulating FN1 degradation (Fata *et al*, 2001). Given these changes, it is possible that ErbB-2 overexpression may alter the adhesive properties of these cells (see below).

### Protein expression correlates with gene expression

**Figure 2:**
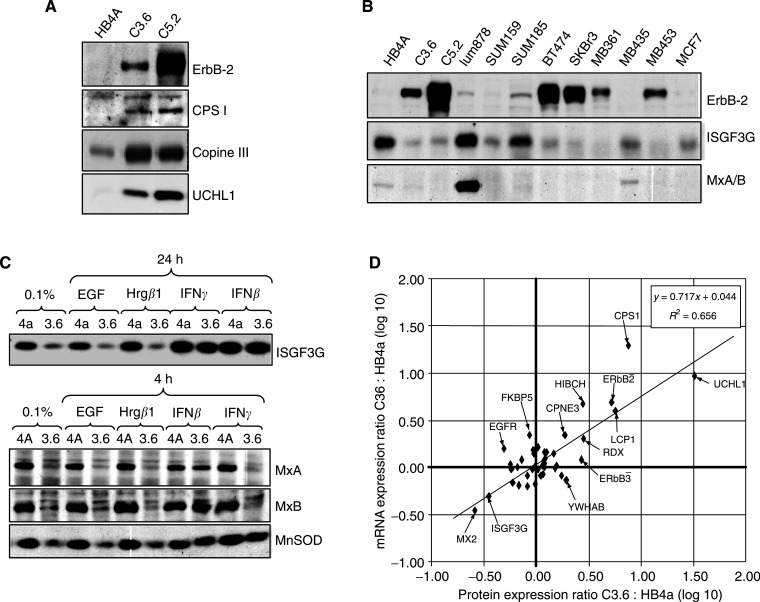
Validation of differential gene expression at the protein level. (**A**) Upregulation of ErbB-2, carbamoyl phosphate synthetase 1 (CPS1I), copine III, ubiquitin C-terminal hydrolase L1 (UCHL1) and cyclin D2 (CCND2) protein in C3.6 and C5.2 ErbB-2-overexpressing HMLECs. Total cell lysates from serum-starved cells were immunoblotted with specific antibodies. The right-hand panel shows UCHL1 levels by immunoblotting in luminal epithelial cells and in breast tumour cell lines. (**B**) Protein expression (by immunoblotting) of ErbB-2, EGFR, ErbB-3, CPS1, Copine III, ISGF3G and MxA in a panel of breast tumour cell lines. (**C**) Effect of EGF, HRG*β*1, IFN*β* and IFN*γ* stimulation on expression of ISGF3G (upper panel), MxA, MxB and MnSOD (lower panel) in HMLECs. (**D**) Correlation between mRNA and protein ratios of 43 genes in serum-starved C3.6 cells, relative to HB4a cells. The mRNA data are derived from the microarray results described in this study, while the protein data are derived from the results of a parallel, quantitative 2D-DIGE study (Gharbi *et al*, 2002) and quantitation of immnuoblotting data (Timms *et al* (2002) and unpublished data).

In order to ascertain whether the observed changes in gene expression translate into similar changes in protein expression, we carried out a comparison of mRNA and protein expression. Protein data were taken from a parallel proteomics study of serum-starved HB4a and C3.6 cells using two-dimensional difference gel electrophoresis (2D-DIGE), and from the quantitation of multiple immunoblotting experiments. The 2D-DIGE analysis identified 30 proteins that were significantly up- or downregulated in response to ErbB-2 overexpression (Gharbi *et al* (2002) and unpublished data). Five upregulated proteins that were represented on the microarrays were also found to be constitutively upregulated in the mRNA analysis. These were: CPS1, LCP1, the metabolic enzyme 3-hydroxyisobutyryl coenzyme A hydrolase (HIBCH), copine III, which may be involved in membrane trafficking (Creutz *et al*, 1998) and ubiquitin C-terminal hydrolase isozyme L1 (UCHL1), a deubiquitinating enzyme whose overexpression has been associated with progression of non-small-cell lung cancers (Hibi *et al*, 1999) and colon cancer invasiveness (Yamazaki *et al*, 2002). The ERM protein radixin (RDX) was also found to be upregulated by 2D-DIGE analysis, but fell just below the two-fold cutoff used in the microarray analysis. The upregulation of several of these gene products, including CCND2, was further confirmed by immunoblotting (Figure 2A

). Notably, protein levels were also increased in another HMLEC clone (C5.2), which expresses even higher levels of ErbB-2. These genes thus appear to be both transcriptionally and translationally upregulated in response to ErbB-2 overexpression.

We further tested the relationship between ErbB-2 expression and the expression of UCHL1, CPS1 and copine III in a panel of breast cancer cell lines. UCHL1 (Figure 2A, right-hand panel) was restricted to luminal epithelial cells (HB4a, C3.6, C5.2 and Lum878), and was not detected in any of the breast tumour cell lines which do not display a luminal epithelial cell morphology. CPS1 protein expression was also restricted to luminal cells, although it was also detected in BT474, which expresses high levels of ErbB-2 and ErbB-3 (Figure 2B). Copine III was expressed in all cell lines, and its expression correlated with both ErbB-3 and ErbB-2 overexpression. In agreement with the microarray analysis, four IFN-inducible genes (ISGF3G, MnSOD and the IFN-regulated viral resistance GTP-binding proteins MxA and MxB) were shown to be significantly downregulated at the protein level (Figure 2B and C). Moreover, expression of ISGF3G and MxA was inversely correlated with ErbB-2 expression in the panel of breast tumour cell lines (Figure 2B). To further examine the regulation of these genes, serum-starved cells were treated with EGF, HRG*β*1, IFN*γ*, or IFN*β*, and expression levels determined by immunoblotting (Figure 2C). While the ErbB receptor ligands had no effect on expression, IFN*γ* treatment resulted in the induction of ISGF3G and MnSOD in the C3.6 cells, and IFN*β* treatment induced the expression of all four proteins. Thus, although the cell lines are responsive to IFN treatment, the basal levels of these IFN-inducible genes are suppressed in response to ErbB-2 overexpression.

An overall comparison of the protein and mRNA ratios for 43 genes showed a statistically significant and high correlation co-efficient of 0.81 (*p*<0.001), and a moderate linear regression (*R*^2^) of 0.66 (*P*<0.001) (Figure 2D). Thus, altered mRNA expression correlates strongly and positively with increased translation and protein expression. The moderate R^2^ value, however, indicates that mRNA level cannot necessarily be used to predict a gene's protein level. To our knowledge, this is the highest correlation between protein and mRNA ratios observed in cells from a higher organism, although we speculate that ErbB-2 overexpression must direct the differential translational regulation and/or protein stability of some genes, rather than their transcription alone. For example, such post-transcriptional regulation would explain the modulated expression of EGFR and YWHAB (Figure 2D), for which mRNA and protein levels do not correlate.

### Genes differentially expressed in response to growth factor treatment

We next examined the genes that were differentially expressed in response to HRG*β*1 stimulation. For brevity, the genes are listed in Tables S2-S5 of Supplementary Data, and we only focus here on several interesting groups that may have an impact on ErbB-2-dependent cellular phenotypes. Of the downregulated genes, cyclin-dependent kinase inhibitor 3 (CDKN3), growth arrest and DNA-damage-inducible *α* (GADD45A) and CDKN2C/p18 are known to be cdk inhibitors (Gyuris *et al*, 1993; Guan *et al*, 1994; Jin *et al*, 2000). Their downregulation thus correlates with the observed hyperactivity of cdk6 and cdk2 in the C3.6 cells, as previously reported (Timms *et al*, 2002), and suggests one mechanism by which ErbB-2 can promote proliferation through enhanced cell cycle progression.

Notably, additional IFN-inducible genes were also downregulated in the C3.6 cells in a HRG*β*1-dependent manner (S3B and S5B). These include MxB, IFN-regulatory factor 3 (IRF3), IRF7, HLA I *α*-chain, death-associated protein kinase 1, tryptophanyl-tRNA synthetase and retinoic acid and IFN-inducible 58 kDa protein. The ISGF3 complex component STAT1 was also downregulated at T18, while the suppressor of cytokine signalling-2 (SOCS-2), a STAT-induced STAT inhibitor (Krebs and Hilton, 2000), was upregulated. These data again support the notion that ErbB-2 overexpression can suppress IFN signalling to downregulate the expression of IFN-inducible genes.

### ErbB-2-dependent modification of HMLEC adhesion

**Figure 3:**
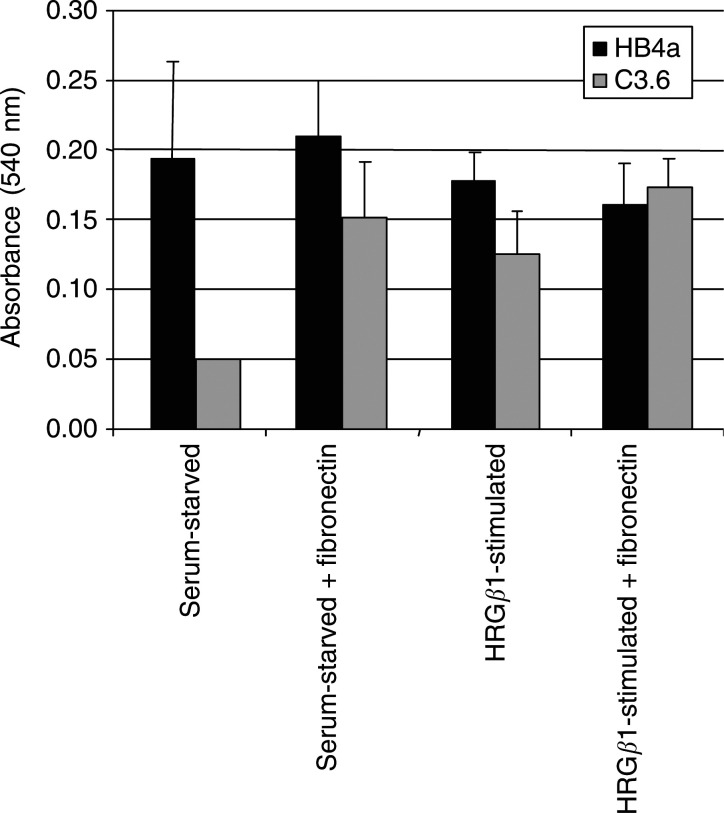
Cell adhesion assays on C3.6 and HB4a in serum-free media, or media supplemented with 1 mM HRG*β*1 as measured by incorporation of MTT into living cells (A_540 nm_). Adhesion onto plastic or fibronectin-coated plates was measured after 1 h. Results are the average of eight replicates, and error bars represent the standard deviation.

A significant number of ECM components or genes involved in ECM modelling, degradation and cell adhesion were also found to be differentially expressed following HRG*β*1 treatment. These include the ECM components fibulin 1 and collagens *α*1(I) and *α*1(VIII), the integrin subunits *α*1, *α*2, *β*4 and *α*V, the proteases cathepsins H and L and matrilysin (MMP7), the protease inhibitor TIMP2, the *β*-galactoside-binding proteins galectins 2 ,7 and 9 and uPAR, the receptor for urokinase plasminogen activator, a serine protease that has been linked to breast cancer through its association with integrins (van der Pluijm *et al*, 2001). Given that we also observed the constitutive downregulation of other genes involved in cellular adhesion (Table 1), we tested the hypothesis that ErbB-2 reduces adhesion and that this can be modulated by HRGβ1 stimulation. Adhesion assays were thus performed under serum-starved conditions and 1 h after HRG*β*1 treatment (Figure 3

). Since the integral ECM component FN1 is constitutively downregulated in C3.6 cells, adhesion was also tested on FN-coated plastic. We found that serum-starved C3.6 cells were significantly less adhesive than HB4a cells on plastic, although their adhesion was increased on FN-coated plastic, or when cells were treated with HRG*β*1 (Figure 3). Moreover, attachment to FN in the presence of HRG*β*1 resulted in a similar level of adhesion for both cell types. These data show that the ErbB-2-dependent expression of these genes does indeed translate to a phenotype of decreased adhesion, which can be partially rescued by FN restoration, or HRG*β*1stimulation, and rescued fully by both treatments.

### Differential regulation of autocrine/paracrine signalling by ErbB-2

**Figure 4:**
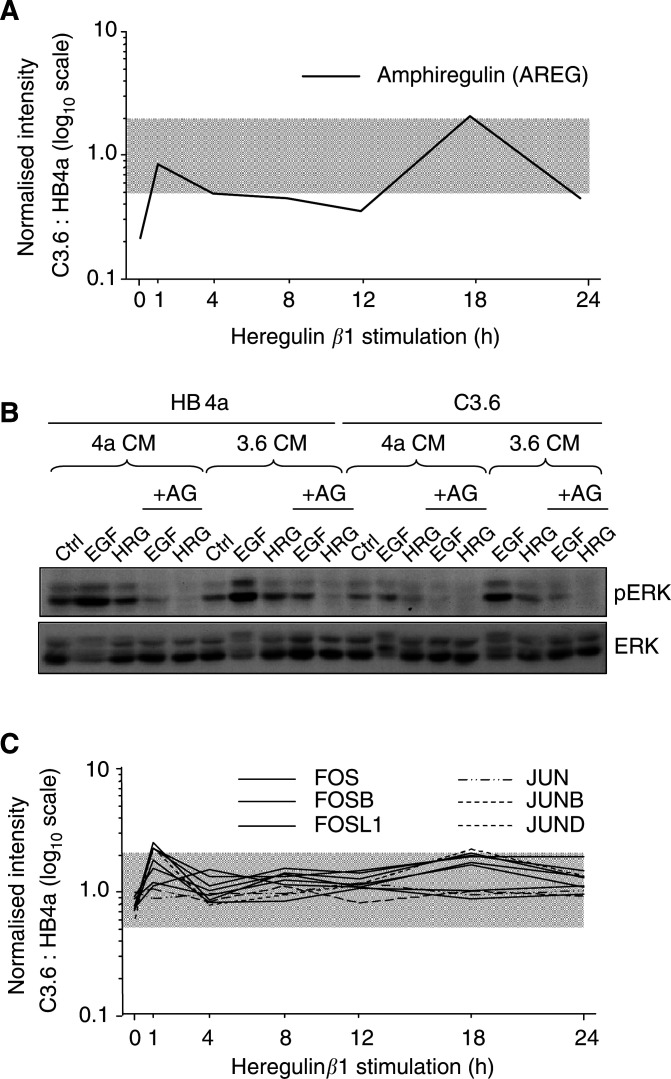
Expression of an autocrine growth factor and components of the AP-1 transcription complex. (**A**) Expression profile (C3.6 : HB4a) of amphiregulin precursor (AREG) across the timecourse of HRG*β*1 stimulation: (**B**) Treatment of serum-starved cells with conditioned media from EGF or HRG*β*1-stimulated cells results in an ErbB-dependent activation of MAPK signalling. Conditioned media (CM) were generated as described in Materials and Methods, and used to stimulate serum-starved and AG-1478-treated HB4a and C3.6 cells for 15 min. MAPK activation was monitored by immunoblotting with antibodies recognising the phosphorylated/activated forms of ERK1 and 2. Blots were reprobed with anti-ERK antibodies to check protein levels. (**C**) HRG*β*1-induced expression profile of Fos and Jun family members (components of the AP-1 transcription complex) in C3.6 *vs* HB4a cells.

The microarray analysis also identified a number of differentially regulated genes involved in autocrine and paracrine responses in breast cancer. These include: amphiregulin (AREG), an autocrine growth factor ligand of EGFR; glypican-1 precursor (GPC1), a modulator of the mitogenic activities of ErbB receptor ligands such as HRG*β*1 and AREG, and frequently upregulated in breast cancer (Matsuda *et al*, 2001); parathyroid hormone-related protein precursor (PTHLH), a signaller of bone remodelling found overexpressed in bone-metastasised breast cancers (Iddon *et al*, 1999); endothelin-2 (EDN2), stanniocalcin 1 (STC1); adrenomedullin precursor (ADM) and secretory granule proteoglycan core protein (PRG1). Of all the genes in the analysis, AREG expression was the most variable across the time course; five-fold downregulated at T0, equivalent expression at T1, two-fold down at T4-12, two-fold up at T18, and two-fold down at T24 (Figure 4A

). These data are indicative of a differential autocrine response following HRG*β*1 stimulation.

To confirm the autocrine secretion of mitogenic ligands, serum-starved HB4a and C3.6 cells were treated with conditioned media (CM) from serum-starved (Ctrl), EGF-, or HRG*β*1-treated cells, and activation of MAPK signalling monitored after 15 min by immunoblotting with a phosphospecific antibody (Figure 4B). This showed that CM from both cell types could trigger MAPK signalling (ERK1/2 activation), with C3.6 CM producing a higher level of activation. Moreover, pretreatment of cells with a potent ErbB kinase inhibitor (AG-1478) significantly inhibited this response (Figure 4B, lanes labelled +AG), supporting the notion that ligands such as AREG are secreted in response to growth factor treatment and can activate autocrine mitogenic signalling through the ErbB receptor system.

Notably, a conspicuous number of downstream mediators of MAPK signalling were also upregulated in the C3.6 cells in to response short-term HRG*β*1 treatment (T1) and again at later timepoints. These genes include components of the AP-1 transcription complex (c-FOS, FOS-B, FOS-related antigen 1, JUN-B (Figure 4C)), the transcription factor c-ETS-2 and the orphan nuclear receptor HMR (Table S4A). This apparent biphasic regulation is suggestive of two waves of transcription as cells respond to HRG*β*1, and their increased expression consistent with a higher mitogenic signalling capacity that correlates with hyperactivation of MAPK signalling, as previously observed in the ErbB-2-overexpressing cells (Timms *et al*, 2002).

## DISCUSSION

The identification of genes that are differentially expressed in ErbB-2-dependent breast cancer is an important step in elucidating the mechanisms of tumorigenesis. To this end, we carried out a parallel microarray and proteomic analysis to investigate ErbB-2- and HRG*β*1-dependent changes in gene expression in a relevant model cell system of breast cancer. This study also addresses whether the differential expression of genes could be involved in generating ErbB-2-dependent cellular phenotypes such as hyperproliferation, mitogen- and anchorage-independent growth.

We firstly report that the correlation between mRNA and protein expression for a subset of 43 genes was very high (0.81) in these cells. This conflicts somewhat with previous reports using human liver (Anderson and Seilhamer, 1997), lung adenocarcinomas (Chen *et al*, 2002) and *S. cerevisiae* (Gygi *et al*, 1999), where lower correlations were observed. This discrepancy may be due to the different cell types used, the different methods of mRNA and protein quantitation used, and/or the subset of genes used for the comparisons. However, the comparison performed here not only helps to validate the findings of the microarray analysis, but also points to the mode of regulation of these differentially regulated genes. Thus, while it appears that the expression of the majority of genes is controlled at the level of transcription in response to ErbB-2 overexpression, a significant minority (∼33%) are regulated post-transcriptionally, in agreement with the reports mentioned above.

Among the genes and proteins shown to display altered expression, six functional groups were identified: genes with roles in cellular metabolism, regulation of cell cycle progression, IFN signalling, cell adhesion, autocrine/paracrine signalling and AP-1-mediated transcription. Interestingly, several of the constitutively deregulated genes identified (S100P, LCP1, vimentin, IGFBP3 and FN1) have been associated with breast tumorigenesis in other studies. This finding not only validates the model cell system used here, but also supports the usefulness of these genes as markers of breast cancer progression. A recent study by Mackay *et al*., 2003 also reported microarray analysis of genes associated with ErbB-2 expression in the HB4a, C3.6, C5.2 model system. The same microarrays were employed as in our study, and 61 genes were reported as significantly up- or downregulated. Consequently, a number of differentially expressed genes identified in the two studies are the same: S100P, CPS1, vimentin (constitutively upregulated), IGFBP3, FN1, SPARC (constitutively downregulated) and 12 other genes found to be differentially expressed at one or more timepoints, or from the protein analysis. This consistency supports the identification of these genes as *bona fide* targets of ErbB-2 overexpression. However, there were several novel gene expression changes that were unique to our study (e.g. CCND2, ISGF3G, UCHL1, LCP1 and CPNE3), and that positively correlated with proteomic changes. The divergence between the two studies is likely to reflect differential data extraction as a result of differential hybridisation, hybridisation artefacts and signal-to-noise ratios across chips and between experiments, but may also reflect the fact that cells were cultured under different conditions.

Our previous study using this cell system demonstrated that enhanced MAPK activation in the ErbB-2-overexpressing cells played a critical role in cell cycle progression and proliferation by promoting cdk6 and cdk2 activity (Timms *et al*, 2002). This was due to a combination of increased cyclin D1 and cdk6 protein expression, and a reduction in the expression level and binding of the cdk inhibitor p27 to cdk2. The present study identifies additional potential effectors of ErbB-2-dependent cell cycle progression, including HRG*β*1-dependent downregulation of three cdk inhibitors and the constitutive upregulation of CCND2 (cyclin D2). Although cyclins are generally considered as positive mediators of mitogen-induced cell cycle progression, it has been shown that loss of CCND2 expression occurs in primary breast tumours (Evron *et al*, 2001). Despite this, expression of CCND2 can reduce the dependence on serum for proliferation of breast epithelial cells, and promote cell cycle progression through activation of cdk2 (Sweeney *et al*, 1997). Thus, a combination of factors appears to drive ErbB-2-dependent cell cycle progression and hence tumorigenic potential in ErbB-2-overexpressing luminal epithelial cells.

A novel and surprising result of this study was the downregulation of multiple IFN-inducible genes in the ErbB-2-overexpressing cells. This downregulation was shown at both the protein level (ISGF3, MxA, MxB and MnSOD) as well as the mRNA level (seven constitutive and a further seven HRG*β*1-dependent genes), and there was a strong inverse correlation between ErbB-2 expression and ISGF3G expression in the panel of breast tumour cell lines used. Since IFN stimulation produced a robust induction of several of these genes (Figure 2), these data imply that ErbB-2 overexpression basally represses IFN signalling in the absence of IFNs. The ISGF3G subunit is a critical component of the ISGF3 transcriptional activator complex for many IFN-inducible genes (Bluyssen *et al*, 1996). We propose that its downregulation could produce the observed suppression of other IFN-inducible genes. IFNs are antiproliferative and can promote apoptosis under certain conditions (reviewed in Stark *et al*, 1998), so the reduced expression of these genes is likely to contribute to the increased proliferative capacity of C3.6 cells. The observed constitutive downregulation of insulin-like growth factor-binding protein 3 (IGFBP3) is also interesting in this context. As well as regulating the circulating levels of IGFs to suppress proliferation, it has been proposed that IFN*γ*'s antiproliferative effect is mediated through IGFBP3 (Katz *et al*, 1999). The modulation of IFN signalling in ErbB-2-overexpressing breast cancers warrants further investigation, and may possibly identify new therapy targets, and/or allow optimisation of current therapies that utilise IFNs to treat patients with cancer (Tossing, 2001).

A significant number of the differentially regulated genes are known modulators of ECM modelling and cellular adhesion. Specifically, FN1, TIMP3 and FBLN2 were all constitutively downregulated, changes which are likely to affect integrin-mediated cellular adhesion. In support of this, we showed that, under serum-starved conditions, the C3.6 cells were less adherent, consistent with ErbB-2 promoting anchorage-independent growth (Harris *et al*, 1999). The apparent rescue of this adhesion phenotype by plating onto fibronectin (Figure 3) substantiates the notion that ErbB-2 suppression of FN1 plays a functional role in anchorage-independent growth and metastasis, as reported previously (Werbajh *et al*, 1998; Ignatoski *et al*, 2000). While our data also suggested that HRG*β*1 can modify adhesion, and hence the potential for invasiveness, the mechanistic details of this modification are unclear and require further detailed investigation.

Finally, the temporal differential expression of AP-1 transcription complex components (Figure 4C) is suggestive of biphasic transcriptional activity following HRG*β*1 stimulation. Our hypothesis is that an immediate-early wave of transcription is induced by HRG*β*1, and that a secondary wave of transcription, mediated via the MAPK pathway, is induced by autocrine/paracrine growth factor production. In support of this, we saw the upregulation of numerous autocrine factors, and further showed that conditioned media from cells treated with either EGF or HRGβ1 was able to activate MAPK signalling when added to serumstarved cells. Moreover, this activation could be blocked by pretreatment of cells with an inhibitor of ErbB receptor kinase activity (AG-1478). This suggests that ErbB-dependent autocrine signalling occurs in these cells (possibly through production of AREG), a process which may contribute to enhanced proliferation.

In summary, it appears that ErbB-2 overexpression produces a combination of increased and temporal MAPK signalling, increased ErbB-related autocrine signalling, increased cdk activity (via upregulation of cyclin D1 and CCND2, and downregulation of cdk inhibitors), inhibition of basal IFN signalling and reduced cellular adhesion. Together, these changes would drive the anchorage-independent proliferation of HMLECs to promote tumorigenesis.
