# Supplementary material for: Cellular responses to ErbB-2 overexpression in human mammary luminal epithelial cells: comparison of mRNA and protein expression
Source: Br J Cancer. 2004 Jan 6;90(1):173–81. doi: 10.1038/sj.bjc.6601458 (PMC2395336; doi:10.1038/sj.bjc.6601458)
Supplement: Supplementary Table 1 [file 90-6601458x1.pdf]

Abbrev	Ensembl Number and Description	Function
	**Constitutively Up-Regulated**	
S100P	ENSG00000163993 S-100P PROTEIN	CA BINDING
CPS1	ENSG00000021826 CARBAMOYL-PHOSPHATE SYNTHASE	METABOLISM
HIBCH	ENSG00000115404 3-HYDROXYISOBUTYRYL-COENZYME A HYDROLASE	METABOLISM
C20orf16	ENSG00000088826 POLYAMINE OXIDASE ISOFORM-3	METABOLISM
	ENSG00000162424 SUCCINATE DEHYDROGENASE	METABOLISM
	ENSG00000130021 GS1 PROTEIN	METABOLISM
FDX1	ENSG00000137714 ADRENODOXIN, MITOCHONDRIAL PRECURSOR	METABOLISM
COX6C	ENSG00000164919 CYTOCHROME C OXIDASE POLYPEPTIDE VIC PRECURSOR	METABOLISM
CCND2	ENSG00000118971 G1/S-SPECIFIC CYCLIN D2	PROLIFERATION
	ENSG00000151465 PROTEIN D123	PROLIFERATION
UCHL1	ENSG00000154277 UBIQUITIN CARBOXYL-TERMINAL HYDROLASE ISOZYME L1	PROTEIN PROCESSING
ERBB2	ENSG00000141736 RECEPTOR PROTEIN-TYROSINE KINASE ERBB-2 PRECURSOR	SIGNALLING
LCP1	ENSG00000136167 L-PLASTIN (LYMPHOCYTE CYTOSOLIC PROTEIN 1)	STRUCTURAL PROTEIN
VIM	ENSG00000026025 VIMENTIN	STRUCTURAL PROTEIN
KLF12	ENSG00000118922 KRUEPPEL-LIKE FACTOR 12	TRANSCRIPTION
TRIM29	ENSG00000137699 ATAXIA-TELANGIECTASIA GROUP D-ASSOCIATED PROTEIN	TRANSCRIPTION
CPNE3	ENSG00000085719 COPINE III	TRANSPORT
SSFA2	ENSG00000138434 SPERM-SPECIFIC ANTIGEN 2	UNKNOWN
	ENSG00000110696 SMALL ACIDIC PROTEIN	UNKNOWN
	ENSG00000077232 CDNA FLJ14741 FIS, CLONE NT2RP3002628	UNKNOWN
	UNIDENTIFIED TRANSCRIPT	UNKNOWN
	**Constitutively Down-Regulated**	
SPARC	ENSG00000113140 SPARC PRECURSOR	CA BINDING
IFITM1	ENSG00000142089 INTERFERON-INDUCED TRANSMEMBRANE PROTEIN 1	IMMUNE RESPONSE*
WNT5A	ENSG00000114251 WNT-5A PROTEIN PRECURSOR	LIGAND
TYMS	ENSG00000080868 THYMIDYLATE SYNTHASE	METABOLISM
OAS1	ENSG00000089127 2'-5'-OLIGOADENYLATE SYNTHETASE 1	METABOLISM*
PAPSS2	ENSG00000148615 3'-PHOSPHOADENOSINE 5'-PHOSPHOSULFATE SYNTHETHASE 2	METABOLISM
CYBA	ENSG00000051523 CYTOCHROME B-245 LIGHT CHAIN	METABOLISM
FTHFD	ENSG00000144908 10-FORMYLTETRAHYDROFOLATE DEHYDROGENASE	METABOLISM
	ENSG00000133700 INTERFERON INDUCED TRANSMEMBRANE PROTEIN	PROLIFERATION*
	ENSG00000137440 HEPARIN BINDING PROTEIN PRECURSOR	PROLIFERATION
	ENSG00000162569 UBIQUITIN CROSS-REACTIVE PROTEIN PRECURSOR	PROTEIN PROCESSING*
KLK8	ENSG00000160327 NEUROPSIN PRECURSOR	PROTEIN PROCESSING
TIMP3	ENSG00000100234 METALLOPROTEINASE INHIBITOR 3 PRECURSOR (TIMP-3)	PROTEIN PROCESSING
TRIP12	ENSG00000153827 THYROID RECEPTOR INTERACTING PROTEIN 12	PROTEIN PROCESSING
SERPINH1	ENSG00000149257 47 KDA HEAT SHOCK PROTEIN PRECURSOR	PROTEIN PROCESSING
PRSS11	ENSG00000166033 SERINE PROTEASE HTRA1 PRECURSOR	PROTEIN PROCESSING
WFDC2	ENSG00000101443 MAJOR EPIDIDYMIS-SPECIFIC PROTEIN E4 PRECURSOR (HE4)	PROTEIN PROCESSING
C1S	ENSG00000126750 PRECURSOR EC 3.4.21.- SERINE PROTEASE	PROTEIN PROCESSING
IGFBP3	ENSG00000146674 INSULIN-LIKE GROWTH FACTOR BINDING PROTEIN 3 PRECURSOR	SIGNALLING*
	ENSG00000101608 MYOSIN REGULATORY LIGHT CHAIN 2, NONSARCOMERIC	STRUCTURAL PROTEIN
FN1	ENSG00000115414 FIBRONECTIN PRECURSOR (FN)	STRUCTURAL PROTEIN
FBLN2	ENSG00000163520 FIBULIN-2 PRECURSOR	STRUCTURAL PROTEIN
ISGF3G	ENSG00000100915 TRANSCRIPTIONAL REGULATOR ISGF3 GAMMA SUBUNIT	TRANSCRIPTION*
KDELR2	ENSG00000136240 ER LUMEN PROTEIN RETAINING RECEPTOR 2	TRANSPORT
	ENSG00000136193	UNKNOWN
IFIT1	ENSG00000152777 IFN-INDUCED PROTEIN WITH TETRATRICOPEPTIDE REPEATS 1	UNKNOWN*
