# Supplementary material for: Cellular responses to ErbB-2 overexpression in human mammary luminal epithelial cells: comparison of mRNA and protein expression
Source: Br J Cancer. 2004 Jan 6;90(1):173–81. doi: 10.1038/sj.bjc.6601458 (PMC2395336; doi:10.1038/sj.bjc.6601458)
Supplement: Supplementary Table 2 [file 90-6601458x2.pdf]

A. Up at T0 only

Systematic	Ratio	StdDev	Abbrev	Ensembl Number and Description	Function
37663_B	2.38	0.49	PKM2	ENSG00000067225 PYRUVATE KINASE, M1 ISOZYME	METABOLISM
147377_A	2.78	0.45	MAOA	ENSG00000094598 AMINE OXIDASE [FLAVIN-CONTAINING] A	METABOLISM
129543_A	2.79	0.50	MAOA	ENSG00000094598 AMINE OXIDASE [FLAVIN-CONTAINING] A	METABOLISM
123474_A	2.34	2.03	SCD	ENSG00000099194 ACYL-COA DESATURASE	METABOLISM
233081_A	2.04	0.30	DHCR24	ENSG00000116133 DIMINUTO-LIKE PROTEIN	METABOLISM
stSG89206	2.30	0.33		ENSG00000128274 ALPHA-4-GALACTOSYLTRANSFERASE	METABOLISM
248683_A	2.07	0.25	GLUD1	ENSG00000148672 GLUTAMATE DEHYDROGENASE 1	METABOLISM
307207_A	2.06	0.36	PGAM2	ENSG00000164708 PHOSPHOGLYCERATE MUTASE, MUSCLE FORM	METABOLISM
50887_B	2.07	0.40	PRDX3	ENSG00000165672 THIOREDOXIN-DEPENDENT PEROXIDE REDUCTASE	METABOLISM
256410_A	2.08	0.51	PRDX3	ENSG00000165672 THIOREDOXIN-DEPENDENT PEROXIDE REDUCTASE	METABOLISM
306186_A	2.17	0.60	DDX5	ENSG00000108654 PROBABLE RNA-DEPENDENT HELICASE P68	NUCLEIC ACID BINDING
123727_A	2.21	0.70	FKBP5	ENSG00000096060 51 KDA FK506-BINDING PROTEIN (FKBP51)	PROTEIN PROCESSING
327682_A	2.64	0.37	ARHGDIB	ENSG00000111348 RHO GDP-DISSOCIATION INHIBITOR 2	STRUCTURAL PROTEIN
190561_A	2.00	0.86	MYO6	ENSG00000112698 MYOSIN VI	STRUCTURAL PROTEIN
278656_A	2.08	0.27	ITGAV	ENSG00000138448 INTEGRIN ALPHA-V PRECURSOR	STRUCTURAL PROTEIN
262862_A	2.27	0.86	BPAG1	ENSG00000151914 BULLOUS PEMPHIGOID ANTIGEN 1	STRUCTURAL PROTEIN
49615_A	2.13	0.81	EIF4EBP2	ENSG00000148730 4E-BINDING PROTEIN 2	TRANSLATION
292806_A	2.47	1.89	CSE1L	ENSG00000124207 IMPORTIN-ALPHA RE-EXPORTER	TRANSPORT
34136_A	2.40	0.48	SORL1	ENSG00000137642 SORTILIN-RELATED RECEPTOR PRECURSOR	TRANSPORT
48758_A	2.18	0.24	SORL1	ENSG00000137642 SORTILIN-RELATED RECEPTOR PRECURSOR	TRANSPORT
stSG89173	2.71	1.80		ENSG00000100258 HYPOTHETICAL PROTEIN 384D8_7	UNKNOWN
357159_B	2.36	1.29		ENSG00000112378 P53-INDUCED PROTEIN PIGPC1	UNKNOWN
41808_B	2.16	0.36		ENSG00000153395 CDNA FLJ12443 FIS, CLONE NT2RM1000186	UNKNOWN
273592_B	2.10	0.60		UNIDENTIFIED TRANSCRIPT	UNKNOWN
stSG89242	2.07	0.61		UNIDENTIFIED TRANSCRIPT	UNKNOWN
stSG89243	2.18	0.79		UNIDENTIFIED TRANSCRIPT	UNKNOWN
1986495_A	2.33	0.93		UNIDENTIFIED TRANSCRIPT	UNKNOWN
stSG89606	2.07	0.75		UNIDENTIFIED TRANSCRIPT	UNKNOWN

B. Down at T0 only

Systematic	Ratio	StdDev	Abbrev	Ensembl Number and Description	Function
308561_A	0.42	0.16	TNNC1	ENSG00000114854 TROPONIN C	CA BINDING
42724_A	0.47	0.13	HPCAL1	ENSG00000115756 VISININ-LIKE PROTEIN 3	CA BINDING
810813_B	0.40	0.09	S100A2	ENSG00000160675 S100 CALCIUM-BINDING PROTEIN A2	CA BINDING
213514_B	0.50	0.16	FCGRT	ENSG00000104870 IGG RECEPTOR FCRN LARGE SUBUNIT P51 PRECURSOR	IMMUNE RESPONSE
262049_B	0.45	0.04	ATP6M	ENSG00000100554 VACUOLAR ATP SYNTHASE SUBUNIT D	METABOLISM
149199_A	0.50	0.11	ATP6A1	ENSG00000114573 VACUOLAR ATP SYNTHASE CATALYTIC SUBUNIT A	METABOLISM
487188_A	0.45	0.21	FTL	ENSG00000087086 FERRITIN LIGHT CHAIN	METAL BINDING
202535_A	0.48	0.04		ENSG00000125144 METALLOTHIONEIN-IK (MT-1K)	METAL BINDING

240803_A	0.49	0.11		ENSG00000125144 METALLOTHIONEIN-IK (MT-1K)	METAL BINDING
111081_A	0.49	0.08	MT1E	ENSG00000159517 METALLOTHIONEIN-IE (MT-1E)	METAL BINDING
232772_A	0.48	0.16	MT1E	ENSG00000159517 METALLOTHIONEIN-IE (MT-1E)	METAL BINDING
274164_A	0.47	0.11	MT1E	ENSG00000159517 METALLOTHIONEIN-IE (MT-1E)	METAL BINDING
293137_A	0.45	0.08	MT1E	ENSG00000159517 METALLOTHIONEIN-IE (MT-1E)	METAL BINDING
297392_A	0.49	0.14	MT1E	ENSG00000159517 METALLOTHIONEIN-IE (MT-1E)	METAL BINDING
66946_A	0.49	0.12	MT1E	ENSG00000159517 METALLOTHIONEIN-IE (MT-1E)	METAL BINDING
125783_A	0.50	0.15	ADARB1	ENSG00000014442 DOUBLE STRANDED RNA SPECIFIC EDITASE 1	NUCLEIC ACID BINDING
293088_A	0.47	0.09		ENSG00000105323 E1B-55KDA-ASSOCIATED PROTEIN	NUCLEIC ACID BINDING
121357_A	0.48	0.18	CDKN3	ENSG00000100526 CYCLIN-DEPENDENT KINASE INHIBITOR 3	PROLIFERATION
115383_A	0.50	0.14	GADD45A	ENSG00000116717 GROWTH ARREST AND DNA-DAMAGE-INDUCIBLE PROTEIN GADD45 ALPHA	PROLIFERATION
415112_A	0.37	0.06	GADD45A	ENSG00000116717 GROWTH ARREST AND DNA-DAMAGE-INDUCIBLE PROTEIN GADD45 ALPHA	PROLIFERATION
132868_A	0.37	0.06	ANXA3	ENSG00000138772 ANNEXIN III	PROLIFERATION
470393_A	0.44	0.06	MMP7	ENSG00000137673 MATRILYSIN PRECURSOR	PROTEIN PROCESSING
789088_A	0.49	0.06	FYN	ENSG00000010810 PROTO-ONCOGENE TYROSINE-PROTEIN KINASE FYN	SIGNALLING
470769_A	0.50	0.15		ENSG00000131435 LIM PROTEIN RIL (REVERSION-INDUCED LIM PROTEIN)	SIGNALLING
356653_A	0.44	0.07	IGFBP6	ENSG00000167779 INSULIN-LIKE GROWTH FACTOR BINDING PROTEIN 6 PRECURSOR	SIGNALLING
stSG89520	0.48	0.16	DGCR6	ENSG00000093101 DGCR6 PROTEIN (DIGEORGE SYNDROME CRITICAL REGION 6)	STRUCTURAL PROTEIN
1533763_A	0.43	0.12	LGALS2	ENSG00000100079 GALECTIN-2	STRUCTURAL PROTEIN
188403_A	0.21	0.09	TAGLN	ENSG00000149591 TRANSGELIN (SMOOTH MUSCLE PROTEIN 22-ALPHA)	STRUCTURAL PROTEIN
358857_A	0.47	0.15	ID1	ENSG00000125968 DNA-BINDING PROTEIN INHIBITOR ID-1 (ID)	TRANSCRIPTION
417759_A	0.48	0.12	TAF10	ENSG00000166337 TRANSCRIPTION INITIATION FACTOR TFIID 30 KDA SUBUNIT	TRANSCRIPTION
741497_A	0.21	0.09	LCN2	ENSG00000148346 NEUTROPHIL GELATINASE-ASSOCIATED LIPOCALIN PRECURSOR (NGAL)	TRANSPORT
32327_A	0.49	0.07	MEA	ENSG00000124733 MALE-ENHANCED ANTIGEN-1 (MEA-1)	UNKNOWN
121728_A	0.49	0.06		ENSG00000126897 ITBA2 PROTEIN (DXS9879E)	UNKNOWN
230060_A	0.43	0.14		ENSG00000126897 ITBA2 PROTEIN (DXS9879E)	UNKNOWN
148677_A	0.41	0.13		ENSG00000167246 PRO2605	UNKNOWN
201843_B	0.43	0.20		ENSG00000167246 PRO2605	UNKNOWN
167165_A	0.48	0.16		UNIDENTIFIED TRANSCRIPT	UNKNOWN
