# Supplementary material for: Cellular responses to ErbB-2 overexpression in human mammary luminal epithelial cells: comparison of mRNA and protein expression
Source: Br J Cancer. 2004 Jan 6;90(1):173–81. doi: 10.1038/sj.bjc.6601458 (PMC2395336; doi:10.1038/sj.bjc.6601458)
Supplement: Supplementary Table 3 [file 90-6601458x3.pdf]

**A. Up at T0 but not T1**

| Time (hours) | 0 | | 1 | | | | |
|---|---|---|---|---|---|---|---|
| **Systematic** | **Ratio** | **StdDev** | **Ratio** | **StdDev** | **Abbrev** | **Ensembl Number and Description** | |
| 120106_C | 2.42 | 0.23 | 1.16 | 0.38 | CASP1 | ENSG00000137752 INTERLEUKIN-1 BETA CONVERTASE PRECURSOR | APOPTOSIS |
| 120106_A | 2.45 | 0.42 | 1.31 | 0.18 | CASP1 | ENSG00000137752 INTERLEUKIN-1 BETA CONVERTASE PRECURSOR | APOPTOSIS |
| 486506_A | 2.08 | 0.29 | 1.24 | 0.24 | CASP1 | ENSG00000137752 INTERLEUKIN-1 BETA CONVERTASE PRECURSOR | APOPTOSIS |
| 37663_B | 2.38 | 0.49 | 1.38 | 0.26 | PKM2 | ENSG00000067225 PYRUVATE KINASE, M1 ISOZYME | METABOLISM |
| 147377_A | 2.78 | 0.45 | 1.58 | 0.28 | MAOA | ENSG00000094598 AMINE OXIDASE | METABOLISM |
| 129543_A | 2.79 | 0.50 | 1.59 | 0.39 | MAOA | ENSG00000094598 AMINE OXIDASE | METABOLISM |
| 123474_A | 2.34 | 2.03 | 1.29 | 1.13 | SCD | ENSG00000099194 ACYL-COA DESATURASE | METABOLISM |
| 36377_A | 2.94 | 0.76 | 0.93 | 0.32 | CPE | ENSG00000109472 CARBOXYPEPTIDASE H PRECURSOR | METABOLISM |
| 142664_A | 2.02 | 0.26 | 1.82 | 0.68 | SIAT4C | ENSG00000110080 CMP-N-AcNEURAMINATE-β-GALACTOSAMIDE-α-2,3-SIALYLTRANSFERASE | METABOLISM |
| 233081_A | 2.04 | 0.30 | 1.39 | 0.26 | DHCR24 | ENSG00000116133 DIMINUTO-LIKE PROTEIN | METABOLISM |
| stSG89206 | 2.30 | 0.33 | 1.49 | 1.67 | | ENSG00000128274 ALPHA-4-GALACTOSYLTRANSFERASE | METABOLISM |
| 322925_B | 2.44 | 0.60 | 1.21 | 0.05 | LDHA | ENSG00000134333 L-LACTATE DEHYDROGENASE A CHAIN | METABOLISM |
| 248683_A | 2.07 | 0.25 | 1.58 | 0.34 | GLUD1 | ENSG00000148672 GLUTAMATE DEHYDROGENASE 1 | METABOLISM |
| 741459_A | 2.26 | 0.25 | 1.83 | 0.39 | ATP5G3 | ENSG00000154518 ATP SYNTHASE LIPID-BINDING PROTEIN | METABOLISM |
| 241305_A | 2.31 | 0.24 | 1.84 | 0.17 | ATP5G3 | ENSG00000154518 ATP SYNTHASE LIPID-BINDING PROTEIN | METABOLISM |
| 222163_A | 2.40 | 0.41 | 1.40 | 0.21 | CKB | ENSG00000166165 CREATINE KINASE, B CHAIN | METABOLISM |
| 307207_A | 2.06 | 0.36 | 1.26 | 0.01 | PGAM2 | ENSG00000164708 PHOSPHOGLYCERATE MUTASE | METABOLSIM |
| 50887_B | 2.07 | 0.40 | 1.57 | 0.39 | PRDX3 | ENSG00000165672 THIOREDOXIN-DEPENDENT PEROXIDE REDUCTASE | METABOLSIM |
| 256410_A | 2.08 | 0.51 | 1.49 | 0.16 | PRDX3 | ENSG00000165672 THIOREDOXIN-DEPENDENT PEROXIDE REDUCTASE | METABOLSIM |
| 123727_A | 2.21 | 0.70 | 1.74 | 0.20 | FKBP5 | ENSG00000096060 51 KDA FK506-BINDING PROTEIN (FKBP51) | NUCLEIC ACID BINDING |
| 306186_A | 2.17 | 0.60 | 1.86 | 0.24 | DDX5 | ENSG00000108654 PROBABLE RNA-DEPENDENT HELICASE P68 | NUCLEIC ACID BINDING |
| 122342_A | 2.04 | 0.38 | 1.18 | 0.30 | ENG | ENSG00000106991 ENDOGLIN PRECURSOR (CD105 ANTIGEN) | PROLIFERATION |
| 143696_A | 2.07 | 0.21 | 1.38 | 0.22 | ENG | ENSG00000106991 ENDOGLIN PRECURSOR (CD105 ANTIGEN) | PROLIFERATION |
| 300647_A | 2.19 | 0.46 | 1.95 | 0.58 | EMP3 | ENSG00000142227 EPITHELIAL MEMBRANE PROTEIN-3 (EMP-3) | PROLIFERATION |
| 1897054_A | 2.37 | 0.61 | 1.48 | 0.25 | PCSK1 | ENSG00000133303 NEUROENDOCRINE CONVERTASE 1 PRECURSOR | PROTEIN PROCESSING |
| 1569654_A | 3.14 | 0.70 | 1.43 | 0.17 | PCSK1 | ENSG00000133303 NEUROENDOCRINE CONVERTASE 1 PRECURSOR | PROTEIN PROCESSING |
| 359705_A | 2.04 | 0.52 | 1.78 | 0.81 | UBE2H | ENSG00000146845 UBIQUITIN-CONJUGATING ENZYME E2-21 KDA | PROTEIN PROCESSING |
| 1131911_A | 2.03 | 0.84 | 1.68 | 0.88 | RAB27A | ENSG00000069974 RAS-RELATED PROTEIN RAB-27A (RAB-27) | SIGNALLING |
| 714018_A | 2.54 | 0.54 | 1.55 | 0.11 | STMN3 | ENSG00000125528 STATHMIN 3 (SCG10-LIKE PROTEIN) | SIGNALLING |
| 214997_A | 2.35 | 0.24 | 1.54 | 0.24 | COL1A1 | ENSG00000108821 COLLAGEN ALPHA 1(I) CHAIN PRECURSOR | STRUCTURAL PROTEIN |
| 327682_A | 2.64 | 0.37 | 1.71 | 0.66 | ARHGDIB | ENSG00000111348 RHO GDP-DISSOCIATION INHIBITOR 2 | STRUCTURAL PROTEIN |
| 201585_A | 2.02 | 0.93 | 1.66 | 1.07 | ARHGDIB | ENSG00000111348 RHO GDP-DISSOCIATION INHIBITOR 2 | STRUCTURAL PROTEIN |
| 190561_A | 2.00 | 0.86 | 1.34 | 0.10 | MYO6 | ENSG00000112698 MYOSIN VI | STRUCTURAL PROTEIN |
| 811096_A | 2.66 | 0.86 | 1.18 | 1.07 | ITGB4 | ENSG00000132470 INTEGRIN BETA-4 PRECURSOR | STRUCTURAL PROTEIN |
| 278656_A | 2.08 | 0.27 | 1.43 | 0.40 | ITGAV | ENSG00000138448 INTEGRIN ALPHA-V PRECURSOR | STRUCTURAL PROTEIN |
| 262862_A | 2.27 | 0.86 | 1.91 | 0.28 | BPAG1 | ENSG00000151914 BULLOUS PEMPHIGOID ANTIGEN 1 | STRUCTURAL PROTEIN |
| 234237_A | 2.15 | 0.36 | 1.48 | 0.20 | | ENSG00000087842 PIRIN | TRANSCRIPTION |
| 789273_A | 2.89 | 0.36 | 1.98 | 0.71 | NAB1 | ENSG00000138386 NGFI-A BINDING PROTEIN 1 | TRANSCRIPTION |

| | | | | | | | |
|---|---|---|---|---|---|---|---|
| 49615_A | 2.13 | 0.81 | 1.54 | 0.31 | EIF4EBP2 | ENSG00000148730 4E-BINDING PROTEIN 2 | TRANSLATION |
| 428916_A | 2.40 | 0.50 | 1.48 | 0.22 | ABCC2 | ENSG00000023839 CANALICULAR MULTISPECIFIC ORGANIC ANION TRANSPORTER 1 | TRANSPORT |
| 52422_B | 4.66 | 1.97 | 1.54 | 0.22 | SLC1A6 | ENSG00000105143 EXCITATORY AMINO ACID TRANSPORTER 4 | TRANSPORT |
| 292806_A | 2.47 | 1.89 | 1.17 | 0.29 | CSE1L | ENSG00000124207 IMPORTIN-ALPHA RE-EXPORTER | TRANSPORT |
| 34136_A | 2.40 | 0.48 | 1.10 | 0.18 | SORL1 | ENSG00000137642 SORTILIN-RELATED RECEPTOR PRECURSOR | TRANSPORT |
| 48758_A | 2.18 | 0.24 | 1.12 | 0.47 | SORL1 | ENSG00000137642 SORTILIN-RELATED RECEPTOR PRECURSOR | TRANSPORT |
| stSG89173 | 2.71 | 1.80 | 1.16 | 1.01 | | ENSG00000100258 HYPOTHETICAL PROTEIN 384D8_7 | UNKNOWN |
| 268412_A | 2.20 | 0.54 | 1.50 | 0.58 | MCF2 | ENSG00000101977 PROTO-ONCOGENE DBL PRECURSOR | UNKNOWN |
| 327407_B | 2.56 | 0.55 | 1.05 | 0.26 | NDRG1 | ENSG00000104419 NDRG1 PROTEIN | UNKNOWN |
| 743299_A | 2.04 | 1.39 | 1.70 | 1.76 | SPAG1 | ENSG00000104450 INFERTILITY-RELATED SPERM PROTEIN | UNKNOWN |
| 357159_B | 2.36 | 1.29 | 1.65 | 0.20 | | ENSG00000112378 P53-INDUCED PROTEIN PIGPC1 | UNKNOWN |
| 37603_B | 2.08 | 0.12 | 1.67 | 0.29 | | ENSG00000134909 KIAA0712 PROTEIN | UNKNOWN |
| stSG89311 | 2.96 | 0.80 | 1.32 | 0.83 | OSBP2 | ENSG00000138962 OSBP-RELATED PROTEIN 4 | UNKNOWN |
| 795557_B | 2.11 | 0.50 | 1.96 | 0.12 | | ENSG00000148671 ADIPOSE MOST ABUNDANT GENE TRANSCRIPT 2 | UNKNOWN |
| 41808_B | 2.16 | 0.36 | 1.19 | 0.24 | | ENSG00000153395 CDNA FLJ12443 FIS, CLONE NT2RM1000186 | UNKNOWN |
| 183053_A | 2.01 | 0.86 | 1.82 | 0.26 | | ENSG00000164369 DC48 | UNKNOWN |
| 196387_A | 2.58 | 1.29 | 1.56 | 0.12 | | ENSG00000166050 MULTIDRUG RESISTANCE ASSOCIATED PROTEIN | UNKNOWN |
| 273592_B | 2.10 | 0.60 | 1.31 | 0.19 | | UNIDENTIFIED TRANSCRIPT | UNKNOWN |
| stSG89242 | 2.07 | 0.61 | 1.07 | 1.04 | | UNIDENTIFIED TRANSCRIPT | UNKNOWN |
| 51927_A | 2.06 | 0.35 | 1.63 | 0.37 | | UNIDENTIFIED TRANSCRIPT | UNKNOWN |
| 124303_A | 2.05 | 0.77 | 1.93 | 0.63 | | UNIDENTIFIED TRANSCRIPT | UNKNOWN |
| stSG89243 | 2.18 | 0.79 | 1.44 | 0.63 | | UNIDENTIFIED TRANSCRIPT | UNKNOWN |
| 282333_A | 2.32 | 0.27 | 1.99 | 0.73 | | UNIDENTIFIED TRANSCRIPT | UNKNOWN |
| 34497_A | 2.14 | 0.27 | 1.43 | 0.42 | | UNIDENTIFIED TRANSCRIPT | UNKNOWN |
| 1986495_A | 2.33 | 0.93 | 1.88 | 0.32 | | UNIDENTIFIED TRANSCRIPT | UNKNOWN |
| 42963_A | 2.02 | 0.25 | 1.04 | 0.36 | | UNIDENTIFIED TRANSCRIPT | UNKNOWN |
| 40060_A | 2.96 | 1.52 | 1.50 | 0.34 | | UNIDENTIFIED TRANSCRIPT | UNKNOWN |
| stSG89606 | 2.07 | 0.75 | 1.15 | 0.11 | | UNIDENTIFIED TRANSCRIPT | UNKNOWN |

**B. Down at T0 but not T1**

| **Time (hours)** | **0** | | **1** | | | | |
|---|---|---|---|---|---|---|---|
| **Systematic** | **Ratio** | **StdDev** | **Ratio** | **StdDev** | **Abbrev** | **Ensembl Number and Description** | |
| 309943_A | 0.43 | 0.15 | 0.70 | 0.31 | TNFAIP3 | ENSG00000118503 TUMOR NECROSIS FACTOR, ALPHA-INDUCED PROTEIN 3 | APOPTOSIS |
| 308561_A | 0.42 | 0.16 | 0.66 | 0.26 | TNNC1 | ENSG00000114854 TROPONIN C, SLOW SKELETAL AND CARDIAC MUSCLES | CALCIUM BINDING |
| 42724_A | 0.47 | 0.13 | 0.63 | 0.06 | HPCAL1 | ENSG00000115756 VISININ-LIKE PROTEIN 3 | CALCIUM BINDING |
| 810813_B | 0.40 | 0.09 | 0.64 | 0.19 | S100A2 | ENSG00000160675 S100 CALCIUM-BINDING PROTEIN A2 | CALCIUM BINDING |
| 341021_A | 0.43 | 0.16 | 0.84 | 0.19 | | ENSG00000008517 NATURAL KILLER CELLS PROTEIN 4 PRECURSOR | IMMUNE RESPONSE |
| 276483_A | 0.30 | 0.07 | 0.58 | 0.53 | OAS1 | ENSG00000089127 2'-5'-OLIGOADENYLATE SYNTHETASE 1 | IMMUNE RESPONSE |
| 213514_B | 0.50 | 0.16 | 0.61 | 0.50 | FCGRT | ENSG00000104870 IGG RECEPTOR FCRN LARGE SUBUNIT P51 PRECURSOR | IMMUNE RESPONSE |
| 346291_A | 0.46 | 0.12 | 0.75 | 0.36 | GBP1 | ENSG00000117228 INTERFERON-INDUCED GUANYLATE-BINDING PROTEIN 1 | IMMUNE RESPONSE |
| 471638_A | 0.35 | 0.12 | 0.84 | 0.42 | MX2 | ENSG00000157596 IFN-REGULATED RESISTANCE GTP-BINDING PROTEIN MXB | IMMUNE RESPONSE |

| | | | | | | | | |
|---|---|---|---|---|---|---|---|---|
| 1568010_A | 0.21 | 0.05 | 0.84 | 0.59 | AREG | ENSG00000109321 | AMPHIREGULIN PRECURSOR | LIGAND |
| 345158_A | 0.48 | 0.18 | 0.57 | 0.23 | PRODH | ENSG00000100033 | PROLINE OXIDASE | METABOLISM |
| 262049_B | 0.45 | 0.04 | 0.56 | 0.21 | ATP6M | ENSG00000100554 | VACUOLAR ATP SYNTHASE SUBUNIT D | METABOLISM |
| 149199_A | 0.50 | 0.11 | 0.81 | 0.36 | ATP6A1 | ENSG00000114573 | VACUOLAR ATP SYNTHASE CATALYTIC SUBUNIT A | METABOLISM |
| 195338_A | 0.36 | 0.05 | 0.52 | 0.38 | TM7SF2 | ENSG00000149809 | SIMILAR TO TRANSMEMBRANE 7 SUPERFAMILY MEMBER 2 | METABOLISM |
| 487188_A | 0.45 | 0.21 | 0.59 | 0.20 | FTL | ENSG00000087086 | FERRITIN LIGHT CHAIN (FERRITIN L SUBUNIT) | METAL BINDING |
| 289536_A | 0.44 | 0.10 | 0.56 | 0.16 | CCS | ENSG00000110592 | COPPER CHAPERONE FOR SUPEROXIDE DISMUTASE | METAL BINDING |
| 125783_A | 0.50 | 0.15 | 0.58 | 0.10 | ADARB1 | ENSG00000014442 | DOUBLE STRANDED RNA SPECIFIC EDITASE 1 | NUCLEIC ACID BINDING |
| 293088_A | 0.47 | 0.09 | 0.51 | 0.19 | | ENSG00000105323 | E1B-55KDA-ASSOCIATED PROTEIN | NUCLEIC ACID BINDING |
| 357239_A | 0.47 | 0.07 | 0.51 | 0.17 | RPA3 | ENSG00000106399 | REPLICATION PROTEIN A 14 KDA SUBUNIT | NUCLEIC ACID BINDING |
| 204299_A | 0.46 | 0.10 | 0.60 | 0.28 | RPA3 | ENSG00000106399 | REPLICATION PROTEIN A 14 KDA SUBUNIT | NUCLEIC ACID BINDING |
| 358857_A | 0.47 | 0.15 | 0.74 | 0.17 | ID1 | ENSG00000125968 | DNA-BINDING PROTEIN INHIBITOR ID-1 (ID) | NUCLEIC ACID BINDING |
| 1533763_A | 0.43 | 0.12 | 0.58 | 0.16 | LGALS2 | ENSG00000100079 | GALECTIN-2 (BETA-GALACTOSIDE-BINDING LECTIN L-14-II) | PROLIFERATION |
| 121357_A | 0.48 | 0.18 | 0.91 | 0.57 | CDKN3 | ENSG00000100526 | CYCLIN-DEPENDENT KINASE INHIBITOR 3 | PROLIFERATION |
| 341752_A | 0.44 | 0.14 | 0.71 | 0.24 | LGALS7 | ENSG00000104796 | GALECTIN-7 (HKL-14) (PI7) | PROLIFERATION |
| 115383_A | 0.50 | 0.14 | 0.63 | 0.25 | GADD45A | ENSG00000116717 | GROWTH ARREST AND DNA-DAMAGE-INDUCIBLE PROTEIN GADD45α | PROLIFERATION |
| 415112_A | 0.37 | 0.06 | 0.63 | 0.10 | GADD45A | ENSG00000116717 | GROWTH ARREST AND DNA-DAMAGE-INDUCIBLE PROTEIN GADD45α | PROLIFERATION |
| 202535_A | 0.48 | 0.04 | 0.64 | 0.17 | | ENSG00000125144 | METALLOTHIONEIN-IK (MT-1K) | PROLIFERATION |
| 240803_A | 0.49 | 0.11 | 0.65 | 0.22 | | ENSG00000125144 | METALLOTHIONEIN-IK (MT-1K) | PROLIFERATION |
| 111081_A | 0.49 | 0.08 | 0.72 | 0.26 | MT1E | ENSG00000159517 | METALLOTHIONEIN-IE | PROLIFERATION |
| 232772_A | 0.48 | 0.16 | 0.77 | 0.31 | MT1E | ENSG00000159517 | METALLOTHIONEIN-IE | PROLIFERATION |
| 274164_A | 0.47 | 0.11 | 0.72 | 0.27 | MT1E | ENSG00000159517 | METALLOTHIONEIN-IE | PROLIFERATION |
| 293137_A | 0.45 | 0.08 | 0.55 | 0.19 | MT1E | ENSG00000159517 | METALLOTHIONEIN-IE | PROLIFERATION |
| 297392_A | 0.49 | 0.14 | 0.69 | 0.27 | MT1E | ENSG00000159517 | METALLOTHIONEIN-IE | PROLIFERATION |
| 66946_A | 0.49 | 0.12 | 0.68 | 0.16 | MT1E | ENSG00000159517 | METALLOTHIONEIN-IE | PROLIFERATION |
| 48256_A | 0.45 | 0.16 | 0.80 | 0.03 | TGM2 | ENSG00000101418 | PROTEIN-GLUTAMINE GAMMA-GLUTAMYLTRANSFERASE | PROTEIN PROCESSING |
| 2113456_A | 0.33 | 0.06 | 0.56 | 0.19 | SLPI | ENSG00000124107 | ANTILEUKOPROTEINASE 1 PRECURSOR | PROTEIN PROCESSING |
| 796471_A | 0.39 | 0.15 | 0.55 | 0.26 | SLPI | ENSG00000124107 | ANTILEUKOPROTEINASE 1 PRECURSOR | PROTEIN PROCESSING |
| 769707_B | 0.29 | 0.15 | 0.76 | 0.57 | KLK10 | ENSG00000129451 | KALLIKREIN 10 PRECURSOR | PROTEIN PROCESSING |
| 470393_A | 0.44 | 0.06 | 0.50 | 0.31 | MMP7 | ENSG00000137673 | MATRILYSIN PRECURSOR | PROTEIN PROCESSING |
| 753407_B | 0.49 | 0.08 | 0.61 | 0.27 | PSMB10 | ENSG00000141099 | PROTEASOME SUBUNIT BETA TYPE 10 PRECURSOR | PROTEIN PROCESSING |
| 346628_B | 0.26 | 0.10 | 0.90 | 0.27 | S100A8 | ENSG00000143546 | CALGRANULIN A (MIGRATION INHIBITORY FACTOR-RELATED PROTEIN 8) | PROTEIN PROCESSING |
| 122383_A | 0.33 | 0.25 | 1.11 | 0.65 | S100A8 | ENSG00000143546 | CALGRANULIN A (MIGRATION INHIBITORY FACTOR-RELATED PROTEIN 8) | PROTEIN PROCESSING |
| 741497_A | 0.21 | 0.09 | 0.95 | 0.30 | LCN2 | ENSG00000148346 | NEUTROPHIL GELATINASE-ASSOCIATED LIPOCALIN PRECURSOR (NGAL) | PROTEIN PROCESSING |
| 741497_C | 0.26 | 0.08 | 0.68 | 0.23 | LCN2 | ENSG00000148346 | NEUTROPHIL GELATINASE-ASSOCIATED LIPOCALIN PRECURSOR (NGAL) | PROTEIN PROCESSING |
| 789088_A | 0.49 | 0.06 | 0.58 | 0.20 | FYN | ENSG00000010810 | PROTO-ONCOGENE TYROSINE-PROTEIN KINASE FYN | SIGNALLING |
| 666997_A | 0.48 | 0.10 | 0.51 | 0.25 | PTPN2 | ENSG00000128772 | PROTEIN-TYROSINE PHOSPHATASE, NON-RECEPTOR TYPE 2 | SIGNALLING |
| 667593_A | 0.41 | 0.05 | 0.50 | 0.31 | PTPN2 | ENSG00000128772 | PROTEIN-TYROSINE PHOSPHATASE, NON-RECEPTOR TYPE 2 | SIGNALLING |
| 470769_A | 0.50 | 0.15 | 0.62 | 0.25 | | ENSG00000131435 | LIM PROTEIN RIL | SIGNALLING |
| 132868_A | 0.37 | 0.06 | 0.68 | 0.19 | ANXA3 | ENSG00000138772 | ANNEXIN III (LIPOCORTIN III) | SIGNALLING |
| 356653_A | 0.44 | 0.07 | 0.87 | 0.64 | IGFBP6 | ENSG00000167779 | INSULIN-LIKE GROWTH FACTOR BINDING PROTEIN 6 PRECURSOR | SIGNALLING |

| | | | | | | | | |
|---|---|---|---|---|---|---|---|---|
| 130774_A | 0.48 | 0.17 | 0.66 | 0.45 | TIMP2 | ENSG00000035862 | METALLOPROTEINASE INHIBITOR 2 PRECURSOR (TIMP-2) | STRUCTURAL PROTEIN |
| stSG89520 | 0.48 | 0.16 | 0.56 | 0.04 | DGCR6 | ENSG00000093101 | DGCR6 PROTEIN | STRUCTURAL PROTEIN |
| 269787_A | 0.47 | 0.16 | 0.98 | 0.16 | L1CAM | ENSG00000102022 | NEURAL CELL ADHESION MOLECULE L1 PRECURSOR | STRUCTURAL PROTEIN |
| 365366_B | 0.49 | 0.07 | 0.51 | 0.04 | RAC1 | ENSG00000136238 | RAS-RELATED C3 BOTULINUM TOXIN SUBSTRATE 1 | STRUCTURAL PROTEIN |
| 138581_A | 0.21 | 0.12 | 0.87 | 0.27 | TAGLN | ENSG00000149591 | TRANSGELIN (SMOOTH MUSCLE PROTEIN 22-ALPHA) | STRUCTURAL PROTEIN |
| 188403_A | 0.21 | 0.09 | 0.99 | 0.31 | TAGLN | ENSG00000149591 | TRANSGELIN (SMOOTH MUSCLE PROTEIN 22-ALPHA) | STRUCTURAL PROTEIN |
| 44373_B | 0.46 | 0.06 | 0.53 | 0.16 | MLP | ENSG00000162528 | MARCKS-RELATED PROTEIN (MAC-MARCKS) | STRUCTURAL PROTEIN |
| 283427_B | 0.46 | 0.19 | 0.71 | 0.58 | IRF7 | ENSG00000070054 | INTERFERON REGULATORY FACTOR 7 (IRF-7) | TRANSCRIPTION |
| 724588_A | 0.48 | 0.32 | 0.56 | 0.09 | ISGF3G | ENSG00000100915 | TRANSCRIPTIONAL REGULATOR ISGF3 GAMMA SUBUNIT | TRANSCRIPTION |
| 1542082_A | 0.38 | 0.07 | 0.71 | 0.62 | SP110 | ENSG00000135899 | NUCLEAR PHOSPHOPROTEIN | TRANSCRIPTION |
| 451695_B | 0.39 | 0.07 | 0.69 | 0.14 | SP110 | ENSG00000135899 | NUCLEAR PHOSPHOPROTEIN | TRANSCRIPTION |
| 665293_A | 0.40 | 0.21 | 0.77 | 0.35 | NR2F2 | ENSG00000150583 | COUP TRANSCRIPTION FACTOR 2 | TRANSCRIPTION |
| 758467_A | 0.35 | 0.17 | 0.56 | 0.43 | NR2F2 | ENSG00000150583 | COUP TRANSCRIPTION FACTOR 2 | TRANSCRIPTION |
| 417759_A | 0.48 | 0.12 | 0.82 | 0.19 | TAF10 | ENSG00000166337 | TRANSCRIPTION INITIATION FACTOR TFIID 30 KDA SUBUNIT | TRANSCRIPTION |
| 125399_A | 0.48 | 0.17 | 0.58 | 0.10 | NAPG | ENSG00000134265 | GAMMA-SOLUBLE NSF ATTACHMENT PROTEIN | TRANSPORT |
| 269139_A | 0.46 | 0.07 | 0.65 | 0.07 | NAPG | ENSG00000134265 | GAMMA-SOLUBLE NSF ATTACHMENT PROTEIN | TRANSPORT |
| 269139_B | 0.44 | 0.11 | 0.74 | 0.29 | NAPG | ENSG00000134265 | GAMMA-SOLUBLE NSF ATTACHMENT PROTEIN | TRANSPORT |
| 321451_A | 0.47 | 0.14 | 0.82 | 0.17 | CRABP2 | ENSG00000143320 | RETINOIC ACID-BINDING PROTEIN II | TRANSPORT |
| 810687_A | 0.33 | 0.11 | 0.88 | 0.41 | CRABP2 | ENSG00000143320 | RETINOIC ACID-BINDING PROTEIN II | TRANSPORT |
| 119530_C | 0.50 | 0.26 | 0.73 | 0.42 | KCNJ15 | ENSG00000157551 | ATP-SENSITIVE INWARD RECTIFIER POTASSIUM CHANNEL 15 | TRANSPORT |
| 120387_B | 0.40 | 0.04 | 0.67 | 0.53 | | ENSG00000017213 | HYPOTHETICAL 61.7 KDA PROTEIN | UNKNOWN |
| 298367_A | 0.49 | 0.10 | 1.35 | 0.40 | TXNIP | ENSG00000117289 | BRAIN-EXPRESSED HHCPA78 HOMOLOG VDUP1 | UNKNOWN |
| 359721_A | 0.38 | 0.08 | 1.39 | 0.38 | TXNIP | ENSG00000117289 | BRAIN-EXPRESSED HHCPA78 HOMOLOG VDUP1 | UNKNOWN |
| 32327_A | 0.49 | 0.07 | 0.79 | 0.44 | MEA | ENSG00000124733 | MALE-ENHANCED ANTIGEN-1 (MEA-1) | UNKNOWN |
| 322869_A | 0.46 | 0.09 | 0.50 | 0.12 | | ENSG00000125505 | BB1 PROTEIN | UNKNOWN |
| 121728_A | 0.49 | 0.06 | 0.58 | 0.28 | | ENSG00000126897 | ITBA2 PROTEIN (DXS9879E) | UNKNOWN |
| 230060_A | 0.43 | 0.14 | 0.61 | 0.50 | | ENSG00000126897 | ITBA2 PROTEIN (DXS9879E) | UNKNOWN |
| 359462_B | 0.32 | 0.12 | 0.57 | 0.40 | | ENSG00000139926 | CDNA FLJ30983 FIS, CLONE HHDPC2000455 | UNKNOWN |
| 325802_A | 0.40 | 0.20 | 0.51 | 0.35 | | ENSG00000163846 | | UNKNOWN |
| 212036_A | 0.44 | 0.10 | 0.51 | 0.10 | | ENSG00000165072 | HYPOTHETICAL 26.4 KDA PROTEIN | UNKNOWN |
| 148677_A | 0.41 | 0.13 | 0.74 | 0.09 | | ENSG00000167246 | PRO2605 | UNKNOWN |
| 201843_B | 0.43 | 0.20 | 1.07 | 0.21 | | ENSG00000167246 | PRO2605 | UNKNOWN |
| 167165_A | 0.48 | 0.16 | 0.83 | 0.35 | | UNIDENTIFIED TRANSCRIPT | | UNKNOWN |
| 343049_A | 0.34 | 0.12 | 0.52 | 0.14 | | UNIDENTIFIED TRANSCRIPT | | UNKNOWN |
| 363063_A | 0.50 | 0.20 | 1.37 | 0.17 | | UNIDENTIFIED TRANSCRIPT | | UNKNOWN |
| 754479_A | 0.44 | 0.15 | 0.60 | 0.21 | | UNIDENTIFIED TRANSCRIPT | | UNKNOWN |
