# Supplementary material for: Cellular responses to ErbB-2 overexpression in human mammary luminal epithelial cells: comparison of mRNA and protein expression
Source: Br J Cancer. 2004 Jan 6;90(1):173–81. doi: 10.1038/sj.bjc.6601458 (PMC2395336; doi:10.1038/sj.bjc.6601458)
Supplement: Supplementary Table 4 [file 90-6601458x4.pdf]

A. Up at T1 but not T0

Time (hours)	0		1				
**Systematic**	**Ratio**	**StdDev**	**Ratio**	**StdDev**	**Abbrev**	**Ensembl Number and Description**	
146758_A	1.76	0.41	2.43	0.50	NNMT	ENSG00000166741 NICOTINAMIDE N-METHYLTRANSFERASE	METABOLISM
485956_A	1.82	0.45	2.26	0.50	ATP5L	ENSG00000167283 ATP SYNTHASE G CHAIN, MITOCHONDRIAL	METABOLISM
322185_A	0.66	0.34	2.28	0.94	PRDX2	ENSG00000167815 PEROXIREDOXIN 2	METABOLISM
428420_B	1.60	0.23	2.35	0.36	SIAT1	ENSG00000073849 CMP-N-AcNEURAMINATE-β-GALACTOSAMIDE-α-2,6-SIALYLTRANSFERASE	PROTEIN PROCESSING
128816_A	1.33	0.20	2.14	0.27	FBXO20	ENSG00000136153 LOMP PROTEIN	PROTEIN PROCESSING
789143_A	1.55	0.35	3.25	1.72	PRKAR2B	ENSG00000005249 CAMP-DEP. PROTEIN KINASE TYPE II-β REGULATORY CHAIN	SIGNALLING
2005924_A	1.75	0.75	2.08	0.63	MATK	ENSG00000007264 MEGAKARYOCYTE-ASSOCIATED TYROSINE-PROTEIN KINASE	SIGNALLING
22711_A	1.09	0.16	2.09	0.43	RPS6KA2	ENSG00000071242 RIBOSOMAL PROTEIN S6 KINASE ALPHA	SIGNALLING
134448_B	0.95	0.24	3.25	4.73	IL1RL1	ENSG00000115602 INTERLEUKIN 1 RECEPTOR-LIKE 1 PRECURSOR (ST2 PROTEIN)	SIGNALLING
207224_A	1.04	0.22	2.72	4.35	IL1RL1	ENSG00000115602 INTERLEUKIN 1 RECEPTOR-LIKE 1 PRECURSOR (ST2 PROTEIN)	SIGNALLING
21531_A	1.29	0.64	2.84	2.36	RGS2	ENSG00000116741 REGULATOR OF G-PROTEIN SIGNALING 2	SIGNALLING
49581_A	1.71	0.63	4.27	2.19	RGS2	ENSG00000116741 REGULATOR OF G-PROTEIN SIGNALING 2	SIGNALLING
222589_A	1.53	0.37	3.75	1.30	DUSP1	ENSG00000120129 DUAL SPECIFICITY PROTEIN PHOSPHATASE 1	SIGNALLING
146239_A	1.34	0.60	3.25	2.10	DUSP1	ENSG00000120129 DUAL SPECIFICITY PROTEIN PHOSPHATASE 1	SIGNALLING
320389_A	1.77	0.51	2.99	0.48	SNF1LK	ENSG00000142178 PROBABLE SERINE/THREONINE PROTEIN KINASE SNF1LK	SIGNALLING
361933_A	1.62	0.50	2.19	0.25		ENSG00000146897 FKSG35	SIGNALLING
666367_A	0.75	0.27	2.24	1.94	SERPINE1	ENSG00000106366 PLASMINOGEN ACTIVATOR INHIBITOR-1 PRECURSOR	STRUCTURAL PROTEIN
193081_A	1.81	0.67	2.26	1.06	RDX	ENSG00000137710 RADIXIN	STRUCTURAL PROTEIN
144708_A	1.55	0.46	2.19	0.38	ZYX	ENSG00000159840 ZYXIN	STRUCTURAL PROTEIN
110503_A	0.77	0.15	2.28	0.40	FOSL1	ENSG00000110518 FOS-RELATED ANTIGEN 1	TRANSCRIPTION
121976_A	0.73	0.44	2.28	0.21	FOS	ENSG00000119606 PROTO-ONCOGENE PROTEIN C-FOS	TRANSCRIPTION
246190_A	0.72	0.40	2.54	0.31	FOS	ENSG00000119606 PROTO-ONCOGENE PROTEIN C-FOS	TRANSCRIPTION
309893_A	1.22	0.22	5.16	2.36	NR4A1	ENSG00000123358 ORPHAN NUCLEAR RECEPTOR HMR	TRANSCRIPTION
222090_A	1.43	0.63	6.28	4.66	NR4A1	ENSG00000123358 ORPHAN NUCLEAR RECEPTOR HMR	TRANSCRIPTION
260303_A	1.85	0.35	2.19	0.28	ETS2	ENSG00000157557 C-ETS-2 PROTEIN	TRANSCRIPTION
153213_A	0.61	0.22	2.31	0.68	JUNB	ENSG00000167817 TRANSCRIPTION FACTOR JUN-B	TRANSCRIPTION
stSG89231	1.51	0.81	2.30	1.42	PSCD4	ENSG00000100055 CYTOHESIN 4	TRANSPORT
171660_A	1.79	0.51	2.25	0.58	APXL	ENSG00000146950 APICAL-LIKE PROTEIN	TRANSPORT
302059_A	1.87	0.22	2.07	0.14		ENSG00000150449 INDUCED PROTEIN	UNKNOWN
110529_A	1.31	0.12	4.11	1.51		ENSG00000167607 HYPOTHETICAL 5.3 KDA PROTEIN	UNKNOWN
428420_A	1.45	0.76	2.00	0.58		UNIDENTIFIED TRANSCRIPT	UNKNOWN
125183_A	1.67	0.27	2.08	0.68		UNIDENTIFIED TRANSCRIPT	UNKNOWN
205090_A	1.38	0.57	2.47	0.57		UNIDENTIFIED TRANSCRIPT	UNKNOWN
249115_A	1.96	0.24	2.12	0.37		UNIDENTIFIED TRANSCRIPT	UNKNOWN
48777_A	1.93	0.33	2.22	0.67		UNIDENTIFIED TRANSCRIPT	UNKNOWN

B. Down at T1 but not T0

**Time (hours**	**0**		**1**				
**Systematic**	**Ratio**	**StdDev**	**Ratio**	**StdDev**	**Abbrev**	**Ensembl Number and Description**	
1071516_A	0.55	0.18	0.40	0.27	EDN2	ENSG00000127129 ENDOTHELIN-2 PRECURSOR (ET-2)	LIGAND
33859_A	0.73	0.16	0.46	0.15	B3GNT6	ENSG00000168047 N-AcLACTOSAMINIDE β–1,3-N-ACETYLGLUCOSAMINYLTRANSFERASE	METABOLISM
29294_A	0.59	0.05	0.50	0.13	RFC1	ENSG00000035928 ACTIVATOR 1 140 KDA SUBUNIT (REPLICATION FACTOR C LARGE SUBUNIT)	NUCLEIC ACID BINDING
364403_A	0.68	0.18	0.45	0.08	CDKN2C	ENSG00000123080 CYCLIN-DEPENDENT KINASE 6 INHIBITOR (P18-INK6)	PROLIFERATION
784820_A	0.54	0.03	0.44	0.46		ENSG00000125505 BB1 PROTEIN	PROLIFERATION
32991_B	0.57	0.14	0.46	0.11	CTSH	ENSG00000103811 CATHEPSIN H PRECURSOR	PROTEIN PROCESSING
308538_A	0.60	0.16	0.43	0.28	CTSH	ENSG00000103811 CATHEPSIN H PRECURSOR	PROTEIN PROCESSING
200814_A	0.62	0.09	0.44	0.25	MME	ENSG00000114802 NEPRILYSIN	PROTEIN PROCESSING
785655_A	0.60	0.04	0.42	0.24	MME	ENSG00000114802 NEPRILYSIN	PROTEIN PROCESSING
302294_A	0.51	0.08	0.27	0.14	PRSS11	ENSG00000166033 SERINE PROTEASE HTRA1 PRECURSOR	PROTEIN PROCESSING
stSG89269	0.73	0.29	0.49	0.17	TIMP3	ENSG00000100234 METALLOPROTEINASE INHIBITOR 3 PRECURSOR (TIMP-3)	PROTEIN PROCESSING
310771_A	0.50	0.05	0.50	0.19	UBL1	ENSG00000116030 UBIQUITIN-LIKE PROTEIN SMT3C PRECURSOR	PROTEIN PROCESSING
758495_A	0.60	0.04	0.49	0.12	UBL1	ENSG00000116030 UBIQUITIN-LIKE PROTEIN SMT3C PRECURSOR	PROTEIN PROCESSING
418399_A	0.87	0.25	0.44	0.36	APBB2	ENSG00000163697 AMYLOID βA4 PRECURSOR PROTEIN-BINDING FAMILY B MEMBER 2	SIGNALLING
346135_A	0.55	0.15	0.48	0.06	GPC1	ENSG00000063660 GLYPICAN-1 PRECURSOR	SIGNALLING
783646_A	0.58	0.20	0.44	0.36	GPC1	ENSG00000063660 GLYPICAN-1 PRECURSOR	SIGNALLING
25499_A	0.76	0.20	0.47	0.17	PLXNA1	ENSG00000114554 NOV/PLEXIN-A1 PROTEIN	SIGNALLING
261756_A	0.94	0.09	0.39	0.31	COL8A1	ENSG00000138462 COLLAGEN ALPHA 1(VIII) CHAIN PRECURSOR	STRUCTURAL PROTEIN
149370_A	0.52	0.08	0.48	0.13	DPPIV	ENSG00000109861 DIPEPTIDYL-PEPTIDASE I PRECURSOR	STRUCTURAL PROTEIN
stSG89192	0.58	0.19	0.45	0.10	FBLN1	ENSG00000077942 FIBULIN 1 ISOFORM C PRECURSOR	STRUCTURAL PROTEIN
272966_A	0.53	0.08	0.48	0.15	MLP	ENSG00000162528 MARCKS-RELATED PROTEIN (MAC-MARCKS)	STRUCTURAL PROTEIN
290867_A	0.55	0.18	0.47	0.32	MLP	ENSG00000162528 MARCKS-RELATED PROTEIN (MAC-MARCKS)	STRUCTURAL PROTEIN
302367_A	1.08	0.11	0.48	0.13	NID	ENSG00000116962 NIDOGEN PRECURSOR	STRUCTURAL PROTEIN
48009_A	0.62	0.13	0.43	0.28		ENSG00000127055 AGRIN PRECURSOR	STRUCTURAL PROTEIN
305455_A	0.51	0.10	0.48	0.19	ISGF3G	ENSG00000100915 TRANSCRIPTIONAL REGULATOR ISGF3 GAMMA SUBUNIT	TRANSCRIPTION
52681_A	0.70	0.14	0.47	0.03	RELB	ENSG00000104856 TRANSCRIPTION FACTOR RELB (I-REL)	TRANSCRIPTION
137021_A	0.61	0.15	0.46	0.14	GABRE	ENSG00000102287 GAMMA-AMINOBUTYRIC-ACID RECEPTOR ε SUBUNIT PRECURSOR	TRANSPORT
123117_A	0.53	0.20	0.46	0.08	KDELR2	ENSG00000136240 ER LUMEN PROTEIN RETAINING RECEPTOR 2	TRANSPORT
768212_A	0.63	0.04	0.45	0.27		ENSG00000149690 PUTATIVE SMALL GTP-BINDING PROTEIN	TRANSPORT
300021_B	0.54	0.13	0.44	0.29	BCL11A	ENSG00000119866 B-CELL LYMPHOMA/LEUKAEMIA 11A EXTRA LONG FORM	UNKNOWN
240712_A	0.54	0.15	0.39	0.16	C4BPB	ENSG00000123843 C4B-BINDING PROTEIN BETA CHAIN PRECURSOR	UNKNOWN
300851_A	0.54	0.09	0.46	0.07		ENSG00000162470 HYPOTHETICAL 9.7 KDA PROTEIN	UNKNOWN
24984_A	0.70	0.29	0.42	0.13		UNIDENTIFIED TRANSCRIPT	UNKNOWN
26156_A	0.68	0.10	0.39	0.46		UNIDENTIFIED TRANSCRIPT	UNKNOWN
153441_A	0.75	0.12	0.49	0.40		UNIDENTIFIED TRANSCRIPT	UNKNOWN
26469_A	0.57	0.25	0.37	0.24		UNIDENTIFIED TRANSCRIPT	UNKNOWN
145340_A	0.82	0.16	0.45	0.23		UNIDENTIFIED TRANSCRIPT	UNKNOWN
