# Supplementary material for: Cellular responses to ErbB-2 overexpression in human mammary luminal epithelial cells: comparison of mRNA and protein expression
Source: Br J Cancer. 2004 Jan 6;90(1):173–81. doi: 10.1038/sj.bjc.6601458 (PMC2395336; doi:10.1038/sj.bjc.6601458)
Supplement: Supplementary Table 5 [file 90-6601458x5.pdf]

A. Up at T18 only

Time (hours) **18**

Systematic	Ratio	StdDev	Abbrev	Ensembl Number and Description	Function
stSG89201	2.23	0.97	BIK	ENSG00000100290 BCL-2 INTERACTING KILLER	APOPTOSIS
259649_A	3.73	2.58	IER3	ENSG00000137331 RADIATION-INDUCIBLE IMMEDIATE-EARLY GENE IEX-1	APOPTOSIS
249092_A	3.16	1.60	IER3	ENSG00000137331 RADIATION-INDUCIBLE IMMEDIATE-EARLY GENE IEX-1	APOPTOSIS
221567_A	3.45	2.38	PHLDA1	ENSG00000139289 PQ-RICH PROTEIN	APOPTOSIS
1674777_A	3.15	2.45	PHLDA1	ENSG00000139289 PQ-RICH PROTEIN	APOPTOSIS
1404774_A	2.35	1.53	PTHLH	ENSG00000087494 PARATHYROID HORMONE-RELATED PROTEIN PRECURSOR	LIGAND
1568010_A	2.07	2.72	AREG	ENSG00000109321 AMPHIREGULIN PRECURSOR	LIGAND
232638_A	2.38	1.19	ADM	ENSG00000148926 ADM PRECURSOR	LIGAND
153589_A	2.05	1.70	STC1	ENSG00000159167 STANNIOCALCIN 1 PRECURSOR	LIGAND
154007_A	2.60	1.33	STC1	ENSG00000159167 STANNIOCALCIN 1 PRECURSOR	LIGAND
272743_A	2.16	1.21	PRG1	ENSG00000122862 SECRETORY GRANULE PROTEOGLYCAN CORE PROTEIN PREC.	LIGAND
323241_A	2.21	1.27		ENSG00000051128 HOMER-3	METABOLISM
307996_B	2.01	0.70	ENO1	ENSG00000074800 ALPHA ENOLASE	METABOLISM
322710_C	2.05	0.47		ENSG00000128524 VACUOLAR ATP SYNTHASE SUBUNIT F	METABOLISM
788143_A	2.02	0.77	SAT	ENSG00000130066 DIAMINE ACETYLTRANSFERASE	METABOLISM
810316_A	2.06	0.32	IDH3A	ENSG00000166411 ISOCITRATE DEHYDROGENASE	METABOLISM
246240_A	2.15	0.59	SRPK1	ENSG00000096063 SERINE KINASE	NUC. ACID BINDING
295132_A	2.08	0.71	SRPK1	ENSG00000096063 SERINE KINASE	NUC. BINDING
42739_A	2.72	0.79	PTP4A1	ENSG00000112245 PROTEIN TYROSINE PHOSPHATASE PTPCAAX1	PROLIFERATION
323643_A	2.14	1.31	EMP1	ENSG00000134531 EPITHELIAL MEMBRANE PROTEIN-1	PROLIFERATION
429813_A	2.01	1.11	SYK	ENSG00000157236 TYROSINE-PROTEIN KINASE SYK	PROLIFERATION
148379_A	2.22	0.81		ENSG00000120390 PROBABLE PYRROLIDONE CARBOXYLATE PEPTIDASE	PROTEIN PROCESSING
35996_A	2.10	1.44		ENSG00000120390 PROBABLE PYRROLIDONE CARBOXYLATE PEPTIDASE	PROTEIN PROCESSING
488061_A	2.56	0.95	PRSS3	ENSG00000010438 TRYPSIN III PRECURSOR	PROTEIN PROCESSING
1846343_A	2.42	1.22	TGM1	ENSG00000092295 PROTEIN-GLUTAMINE GAMMA-GLUTAMYLTRANSFERASE K	PROTEIN PROCESSING
360874_B	2.65	1.93	TFPI2	ENSG00000105825 TISSUE FACTOR PATHWAY INHIBITOR 2 PRECURSOR	PROTEIN PROCESSING
1336876_A	2.13	1.64	ADAM8	ENSG00000108067 ADAM 8 PRECURSOR	PROTEIN PROCESSING
230382_B	2.48	0.43	F12	ENSG00000131187 COAGULATION FACTOR XII PRECURSOR	PROTEIN PROCESSING
252292_B	2.07	0.31	CTSL	ENSG00000135047 CATHEPSIN L PRECURSOR	PROTEIN PROCESSING
322441_A	2.10	0.57	PLAUR	ENSG00000011422 UROKINASE PLASMINOGEN ACTIVATOR SURFACE RECEPTOR	SIGNALLING
22790_B	2.24	1.72	PLAUR	ENSG00000011422 UROKINASE PLASMINOGEN ACTIVATOR SURFACE RECEPTOR	SIGNALLING
252443_A	2.80	0.67	DUSP4	ENSG00000120875 DUAL SPECIFICITY PROTEIN PHOSPHATASE 4	SIGNALLING
33285_A	2.20	0.63	DUSP5	ENSG00000138166 DUAL SPECIFICITY PROTEIN PHOSPHATASE 5	SIGNALLING
40851_A	3.78	2.04	DUSP6	ENSG00000139318 DUAL SPECIFICITY PROTEIN PHOSPHATASE 6	SIGNALLING
754699_A	2.13	0.80	GNG4	ENSG00000162886 GUANINE NUCLEOTIDE-BINDING PROTEIN	SIGNALLING

38769_A	2.32	1.24	ABLIM	ENSG00000099204 ACTIN-BINDING DOUBLE-ZINC-FINGER PROTEIN	STRUCT. PROTEIN
418128_A	2.30	0.69	MID1	ENSG00000101871 MIDLINE 1 PROTEIN	STRUCT. PROTEIN
158256_A	2.64	1.36	ITGB4	ENSG00000132470 INTEGRIN BETA-4 PRECURSOR	STRUCT. PROTEIN
714132_A	2.42	1.83	ITGB4	ENSG00000132470 INTEGRIN BETA-4 PRECURSOR	STRUCT. PROTEIN
212078_A	2.62	1.18		ENSG00000152684 INTEGRIN ALPHA-1	STRUCT. PROTEIN
50182_A	2.27	1.05	ITGA2	ENSG00000164171 INTEGRIN ALPHA-2 PRECURSOR	STRUCT. PROTEIN
811740_A	3.52	2.08	ITGA2	ENSG00000164171 INTEGRIN ALPHA-2 PRECURSOR	STRUCT. PROTEIN
363387_A	3.32	2.87		ENSG00000102804 REGULATORY PROTEIN TSC-22	TRANSCRIPTION
67043_A	2.28	0.78	EPAS1	ENSG00000116016 ENDOTHELIAL PAS DOMAIN PROTEIN 1	TRANSCRIPTION
32120_A	2.22	0.63	EPAS1	ENSG00000116016 ENDOTHELIAL PAS DOMAIN PROTEIN 1	TRANSCRIPTION
1634407_B	2.25	1.22	FOS	ENSG00000119606 PROTO-ONCOGENE PROTEIN C-FOS	TRANSCRIPTION
668950_B	3.04	2.30	SOCS2	ENSG00000120833 STAT INDUCED STAT INHIBITOR-2	TRANSCRIPTION
667951_A	2.50	1.66	SOCS2	ENSG00000120833 STAT INDUCED STAT INHIBITOR-2	TRANSCRIPTION
141674_B	2.02	0.43	TIF1	ENSG00000122779 TRANSCRIPTION INTERMEDIARY FACTOR 1-ALPHA	TRANSCRIPTION
245100_A	2.06	0.62	GTF2A2	ENSG00000140307 TRANSCRIPTION INITIATION FACTOR IIA GAMMA CHAIN	TRANSCRIPTION
38048_A	2.57	1.43	SRPX	ENSG00000101955 SUSHI REPEAT-CONTAINING PROTEIN SRPX PRECURSOR	UNKNOWN
784928_A	2.61	0.45		ENSG00000102359 SUSHI-REPEAT PROTEIN	UNKNOWN
245473_A	2.00	0.53		ENSG00000123124 WWP1 (FRAGMENT)	UNKNOWN
240833_A	2.31	1.54		ENSG00000130508 MYELOBLAST KIAA0230	UNKNOWN
265370_A	2.01	0.41		ENSG00000162861 HYPOTHETICAL 11.8 KDA PROTEIN	UNKNOWN
381255_A	2.64	0.81		UNIDENTIFIED TRANSCRIPT	UNKNOWN
363590_C	2.04	0.60		UNIDENTIFIED TRANSCRIPT	UNKNOWN
173674_B	2.50	2.50		UNIDENTIFIED TRANSCRIPT	UNKNOWN
359849_A	2.01	1.06		UNIDENTIFIED TRANSCRIPT	UNKNOWN

B. Down at T18 only

**Time (hours)**	**18**				
**Systematic**	**lormalize**	**dDev Nor**	**Common**	**Description**	**Function**
809715_A	0.41	0.06	DAPK1	ENSG00000148075 DEATH-ASSOCIATED PROTEIN KINASE 1	APOPTOSIS
258822_A	0.47	0.09	NGFRAP1	ENSG00000166681 P75NTR-ASSOCIATED CELL DEATH EXECUTOR	APOPTOSIS
300986_A	0.47	0.08	HLA-E	ENSG00000137361 HLA CLASS I HISTOCOMPATIBILITY ANTIGEN, ALPHA CHAIN	IMMUNE RESPONSE
127187_A	0.33	0.14	CRAT	ENSG00000095321 CARNITINE O-ACETYLTRANSFERASE	METABOLISM
stSG89523	0.37	0.21	PRODH	ENSG00000100033 PROLINE OXIDASE	METABOLISM
stSG89234	0.26	0.14	APOL1	ENSG00000100342 APOLIPOPROTEIN L1 PRECURSOR	METABOLISM
248870_A	0.45	0.07	NEU1	ENSG00000111997 SIALIDASE 1 PRECURSOR	METABOLISM
714494_A	0.46	0.23	ATP6B1	ENSG00000116039 VACUOLAR ATP SYNTHASE SUBUNIT B, KIDNEY ISOFORM	METABOLISM
323618_B	0.44	0.04	BTN3A3	ENSG00000124561 BUTYROPHILIN (BUTYROPHILIN, SUBFAMILY 3, MEMBER A3)	METABOLISM
346798_A	0.48	0.20	ALDH3B2	ENSG00000132746 ALDEHYDE DEHYDROGENASE 8	METABOLISM

268248_B	0.46	0.24	WARS	ENSG00000140105	TRYPTOPHANYL-TRNA SYNTHETASE	METABOLISM
306144_A	0.44	0.11		ENSG00000163484	GLUCOSYLCERAMIDASE PRECURSOR	METABOLISM
39682_A	0.48	0.09	RFC1	ENSG00000035928	ACTIVATOR 1 140 KDA SUBUNIT	NUC. ACID BINDING
725276_A	0.48	0.17	MCM6	ENSG00000076003	DNA REPLICATION LICENSING FACTOR MCM6 (P105MCM)	NUC. ACID BINDING
51092_A	0.50	0.09	HDAC6	ENSG00000094631	HISTONE DEACETYLASE 6 (HD6)	NUC. ACID BINDING
137526_A	0.48	0.11		ENSG00000104889	RIBONUCLEASE HI LARGE SUBUNIT	NUC. ACID BINDING
299432_A	0.49	0.09		ENSG00000104889	RIBONUCLEASE HI LARGE SUBUNIT	NUC. ACID BINDING
795562_B	0.48	0.20	SMC4L1	ENSG00000113810	CHROMOSOME-ASSOCIATED POLYPEPTIDE-C	NUC. ACID BINDING
138810_A	0.49	0.11	SSA1	ENSG00000132109	52 KDA RO PROTEIN (SJOGREN SYNDROME TYPE A ANTIGEN	NUC. ACID BINDING
789040_A	0.46	0.18	FYN	ENSG00000010810	PROTO-ONCOGENE TYROSINE-PROTEIN KINASE FYN	PROLIFERATION
725766_A	0.28	0.17	LGALS9	ENSG00000019755	GALECTIN-9 (HOM-HD-21) (ECALECTIN)	APOPTOSIS
810124_A	0.50	0.24	PAFAH1B3	ENSG00000079462	PLATELET-ACTIVATING FACTOR ACETYLHYDROLASEIBγSUB.	PROLIFERATION
359747_A	0.37	0.13	LGALS7	ENSG00000104796	GALECTIN-7 (HKL-14) (PI7)	PROLIFERATION
364964_A	0.37	0.30	LGALS7	ENSG00000104796	GALECTIN-7 (HKL-14) (PI7)	PROLIFERATION
291057_A	0.42	0.05	CDKN2C	ENSG00000123080	CYCLIN-DEPENDENT KINASE 6 INHIBITOR (P18-INK6)	PROLIFERATION
299047_A	0.46	0.09	PRCP	ENSG00000137509	LYSOSOMAL PRO-X CARBOXYPEPTIDASE PRECURSOR	PROTEIN PROCESSING
142418_A	0.48	0.13	USP11	ENSG00000102226	UBIQUITIN CARBOXYL-TERMINAL HYDROLASE 11	PROTEIN PROCESSING
51818_A	0.47	0.03	UBL1	ENSG00000116030	UBIQUITIN-LIKE PROTEIN SMT3C PRECURSOR	PROTEIN PROCESSING
29687_A	0.49	0.06	PEPD	ENSG00000124299	XAA-PRO DIPEPTIDASE	PROTEIN PROCESSING
1147278_A	0.47	0.10	CAPN5	ENSG00000149260	CALPAIN-LIKE PROTEASE	PROTEIN PROCESSING
242008_B	0.27	0.07	UBE2L6	ENSG00000156587	UBIQUITIN-CONJUGATING ENZYME E2-18 KDA	PROTEIN PROCESSING
stSG89435	0.48	0.21	RABL2B	ENSG00000079974	RAB-LIKE PROTEIN 2A	SIGNALLING
49303_A	0.46	0.08	PPP2R2B	ENSG00000156475	SERINE/THREONINE PROTEIN PHOSPHATASE 2A	SIGNALLING
1551330_A	0.48	0.12	SMARCA3	ENSG00000071794	SWI/SNF-REL. ACTIN-DEP. REG. OF CHROMATIN A3	TRANSCRIPTION
144020_A	0.49	0.21	MEIS3	ENSG00000105419	HOMEOBOX PROTEIN MEIS3	TRANSCRIPTION
136252_A	0.41	0.08	GATA3	ENSG00000107485	TRANS-ACTING T-CELL SPECIFIC TRANSCR. FACTOR GATA-3	TRANSCRIPTION
26599_A	0.48	0.17	STAT1	ENSG00000115415	SIGNAL TRANSDUCER AND ACTIVATOR OF TRANSCRIPTION 1	TRANSCRIPTION
299497_B	0.38	0.16	TXNIP	ENSG00000117289	THIOREDOXIN INTERACTING PROTEIN	TRANSCRIPTION
34378_A	0.48	0.13	IRF3	ENSG00000126456	INTERFERON REGULATORY FACTOR 3 (IRF-3)	TRANSCRIPTION
364448_A	0.49	0.27	IKKE	ENSG00000143466	INHIBITOR OF NUCLEAR FACTOR κ-B KINASE ε–SUB.	TRANSCRIPTION
150102_A	0.49	0.13	TLOC1	ENSG00000008952	TRANSLOCATIONAL PROTEIN-1	TRANSPORT
24678_A	0.38	0.27	SLC1A3	ENSG00000079215	EXCITATORY AMINO ACID TRANSPORTER 1	TRANSPORT
38755_A	0.35	0.15	GABRE	ENSG00000102287	γ-AMINOBUTYRIC-ACID RECEPTOR ε-SUBUNIT PRECURSOR	TRANSPORT
25433_A	0.47	0.14	ATP1B1	ENSG00000143153	SODIUM/POTASSIUM-TRANSPORTING ATPASE β-1 CHAIN	TRANSPORT
51782_A	0.46	0.09	TAP1	ENSG00000164643	ANTIGEN PEPTIDE TRANSPORTER	TRANSPORT
179640_A	0.42	0.08	NISCH	ENSG00000010322	NISCHARIN; IMIDAZOLINE RECEPTOR CANDIDATE	UNKNOWN
41288_A	0.35	0.06	TNFAIP2	ENSG00000100660	TUMOR NECROSIS FACTOR, ALPHA-INDUCED PROTEIN 2	UNKNOWN
809923_A	0.46	0.20	TNFAIP2	ENSG00000100660	TUMOR NECROSIS FACTOR, ALPHA-INDUCED PROTEIN 2	UNKNOWN

306154_A	0.43	0.40	SAMHD1	ENSG00000101347 HYPOTHETICAL 72.1 KDA PROTEIN	UNKNOWN
40582_A	0.48	0.19	BAI2	ENSG00000121753 BRAIN-SPECIFIC ANGIOGENESIS INHIBITOR 2 PRECURSOR	UNKNOWN
50086_A	0.40	0.13	BAI2	ENSG00000121753 BRAIN-SPECIFIC ANGIOGENESIS INHIBITOR 2 PRECURSOR	UNKNOWN
129334_A	0.42	0.15		ENSG00000124006 THYRO1000072 PROTEIN	UNKNOWN
260802_A	0.40	0.14		ENSG00000152778 RETINOIC ACID- AND IFN-INDUCIBLE 58 KDA PROTEIN	UNKNOWN
45710_A	0.46	0.25		ENSG00000159164 KIAA0736 PROTEIN	UNKNOWN
44736_A	0.36	0.03		ENSG00000159164 KIAA0736 PROTEIN	UNKNOWN
268853_B	0.48	0.15		UNIDENTIFIED TRANSCRIPT	UNKNOWN
51768_B	0.47	0.07		UNIDENTIFIED TRANSCRIPT	UNKNOWN
193377_A	0.49	0.10		UNIDENTIFIED TRANSCRIPT	UNKNOWN
344984_A	0.43	0.27		UNIDENTIFIED TRANSCRIPT	UNKNOWN
32170_A	0.49	0.15		UNIDENTIFIED TRANSCRIPT	UNKNOWN
810137_A	0.49	0.23		UNIDENTIFIED TRANSCRIPT	UNKNOWN
stSG89552	0.46	0.27		UNIDENTIFIED TRANSCRIPT	UNKNOWN
